# Circulating Exosomal MicroRNA Profiles Associated with Heavy Metal Exposure and Short Stature in Children

**DOI:** 10.3390/ijms27031230

**Published:** 2026-01-26

**Authors:** Min Won Shin, Heeji Kim, Seongho Ryu, Shin-Hye Kim

**Affiliations:** 1 Department of Pediatrics, Inje University Sanggye Paik Hospital, Seoul 01757, Republic of Korea; s5727@paik.ac.kr; 2 Soonchunhyang Institute of Medi-Bio Science (SIMS), Soonchunhyang University, Cheonan 31151, Republic of Korea; khjee1932@naver.com

**Keywords:** heavy metals, Pb, As, Hg, microRNA, short stature, idiopathic short stature, growth hormone deficiency, endochondral ossification

## Abstract

Heavy metal exposure is increasingly linked to impaired childhood growth, but the biological mechanisms are poorly understood. Here, we assessed associations between heavy metal exposure and growth impairment (idiopathic short stature [ISS] and growth hormone deficiency [GHD]) in 36 children (24 cases, 12 controls, males 41.7%), identifying related alterations in circulating exosomal miRNAs. Blood/urine concentrations of nine metals, including Pb, As, and Hg were measured, and serum exosomal miRNAs were profiled via sequencing. Elevated heavy metal exposure was associated with significantly increased proportions of ISS and GHD. Specifically, high blood Pb was associated with ISS (*p* = 0.01) and high urinary As with overall short stature (*p* = 0.03). Elevated urinary Hg showed a marginal association with GHD (*p* = 0.07). Differentially expressed miRNAs were identified: hsa-miR-4488 was downregulated in high-Pb and ISS groups, whereas hsa-miR-133a-3p and hsa-miR-4516 were upregulated in high urinary Hg/As and GHD groups. Predicted targets of these miRNAs involved growth hormone (GH)–insulin-like growth factor-1 (IGF-1) signaling and endochondral ossification. In conclusion, Pb, As, and Hg exposures were associated with impaired growth in children. The dysregulation of related miRNAs suggests biological mechanisms involving both local growth-plate dysfunction and GH-IGF1 signaling disruption.

## 1. Introduction

Childhood growth is a sensitive health indicator and is influenced by genetics, nutrition, endocrine regulation, and environmental exposures [1]. Globally, short stature affects 3–5% of children, with idiopathic short stature (ISS) and growth hormone (GH) deficiency (GHD) being the two main diagnoses [2,3]. Environmental toxicants, such as heavy metals, are increasingly recognized as potential contributors to growth impairment [4,5].

Heavy metals are ubiquitous environmental pollutants with well-documented endocrine-disrupting activity [4,6]. Mechanistically, they generate reactive oxygen species, impair mitochondrial bioenergetics, disrupt calcium and zinc homeostasis, and interfere with hormonal signaling at multiple levels, including GH secretion and insulin-like growth factor-1 (IGF-1) synthesis and function [7,8,9]. Experimental models have revealed direct effects on endochondral ossification—with altered chondrocyte proliferation and hypertrophy—extracellular matrix production, and angiogenesis [10,11]. Collectively, these findings suggest that early-life metal exposure may contribute to growth impairment.

However, human studies on the topic have shown substantial heterogeneity. While several cohort studies have linked childhood exposure to heavy metals such as lead (Pb), arsenic (As), and cadmium (Cd) with modest reductions in height z-scores [4,5,12,13], findings are inconsistent, reflecting differences in exposure metrics (blood vs. urine), exposure windows, timeframes of biomonitoring, and population context [14,15,16]. Moreover, most prior studies evaluated growth based on continuous anthropometry in general populations rather than focusing on clinically defined growth failure. Consequently, the contribution of environmental heavy metals to severe, clinically adjudicated disorders such as ISS and GHD remains insufficiently characterized.

Epigenetic mechanisms provide a biologically credible route through which environmental toxicants may influence endocrine and skeletal biology. MicroRNAs (miRNAs)—small non-coding RNAs that post-transcriptionally repress target transcripts—are integral to growth-plate physiology and GH/IGF signaling; they coordinate chondrocyte lineage commitment, proliferation, hypertrophic maturation, and matrix remodeling and modulate components of the hypothalamic–pituitary–hepatic axis (e.g., GHR/IGF-1 signaling) [17,18]. Circulating miRNAs are detectable in blood, where they are protected from degradation within extracellular vesicles or Argonaute-containing complexes. When pre-analytical handling is controlled, their profiles are sufficiently stable to serve as minimally invasive biomarkers that can reflect tissue states [17]. Experimental and translational studies, including in pregnancy cohorts, have shown that toxic metals perturb miRNA expression and downstream pathways associated with oxidative stress, apoptosis, and differentiation, supporting the biological plausibility of exposure-responsive miRNA signatures [19,20]. However, pediatric human studies concurrently quantifying metal exposures, assessing clinically adjudicated growth failure, and profiling circulating miRNAs are scarce.

To address these gaps, we conducted a cross-sectional pilot study in Korean children to examine the relationship between heavy metal exposure and growth impairment defined as short stature, ISS, or GHD. We quantified nine toxic metals/metalloids, including Pb, As, Cd, mercury (Hg), nickel (Ni), thallium (Tl), antimony (Sb), aluminum (Al), and cesium (Cs), in blood and urine and evaluated their associations with diagnostic categories of growth disorders. Further, we profiled circulating miRNAs to identify exposure-related signatures and explored the putative biological relevance of differentially expressed miRNAs (DEmiRNAs) through target-gene prediction and pathway enrichment with a focus on endochondral ossification and the hypothalamic–pituitary–GH axis.

## 2. Results

### 2.1. Participant Characteristics

Table 1 summarizes the characteristics of the 36 participants (ISS N = 13; GHD N = 11; controls N = 12). Sex distribution and chronological age were comparable across the groups (*p* = 0.915, 0.830, respectively). Bone age delay (CA − BA) was the greatest in the GHD group (overall *p* = 0.003; GHD vs. control significant) and intermediate in the ISS group. Growth-related indices clearly separated cases from controls: height standard deviation score (SDS) and weight SDS were markedly reduced in both ISS and GHD cases vs. controls (*p* < 0.001). Serum IGF-1 concentrations and IGF-1 SDS were also significantly lower in both patient groups than in controls (*p* < 0.001). No pairwise differences between ISS and GHD reached statistical significance after Dunn–Bonferroni correction.

### 2.2. Heavy Metal Exposure Levels in Study Participants

The distributions of blood and urine heavy metal concentrations are provided in Table S1 and Figure S2. All blood analytes (Hg, Pb, As, Al, and Cs) were quantifiable in 100% of the samples. In creatinine-normalized urine, quantifiable concentrations were observe in 100% of samples for Hg, As, Al, Cs, Sb, Tl, and Ni; in 91.7% for Pb; and in 69.4% for Cd (Table S1). A correlation heatmap of blood and urine concentrations of the nine metals is shown in Figure S1. Within-matrix correlations predominated: urinary biomarkers formed a dense cluster of positive associations, whereas blood metals exhibited fewer and weaker correlations, mainly between Hg and Pb. Cross-matrix (blood–urine) correlations were sparse and rather weak; only urinary As and blood As were significantly correlated. Comparison of blood and urine heavy metal and metalloid concentrations in study subjects is presented in Table S2.

### 2.3. Associations Between High-Level Metal Exposure and Short Stature Phenotypes

Table 2 presents the proportions of short stature phenotypes across median-dichotomized exposure categories (<median vs. ≥median) for each measured metal. Elevated blood Pb levels were associated with increased proportions of overall short stature (83.3% vs. 50.0%; *p* = 0.034) and ISS (55.6% vs. 16.7%; *p* = 0.015), whereas no association was observed for GHD. Elevated urinary As levels were associated with an increased proportion of overall short stature (83.3% vs. 50.0%; *p* = 0.034), whereas the proportions of ISS (44.4% vs. 27.8%) and GHD (38.9% vs. 22.2%) were increased, but not significantly. For urinary Hg, the proportion of GHD was borderline higher in the high-exposure than in the low-exposure group (44.4% vs. 16.7%, respectively; *p* = 0.070); however, the difference was not statistically significant. No statistically significant associations were observed with overall short stature or ISS. The other metals (Al, Cs, Cd, Sb, Tl, and Ni in either matrix) showed no significant association with the proportion of any short stature phenotype.

**Figure 1:**
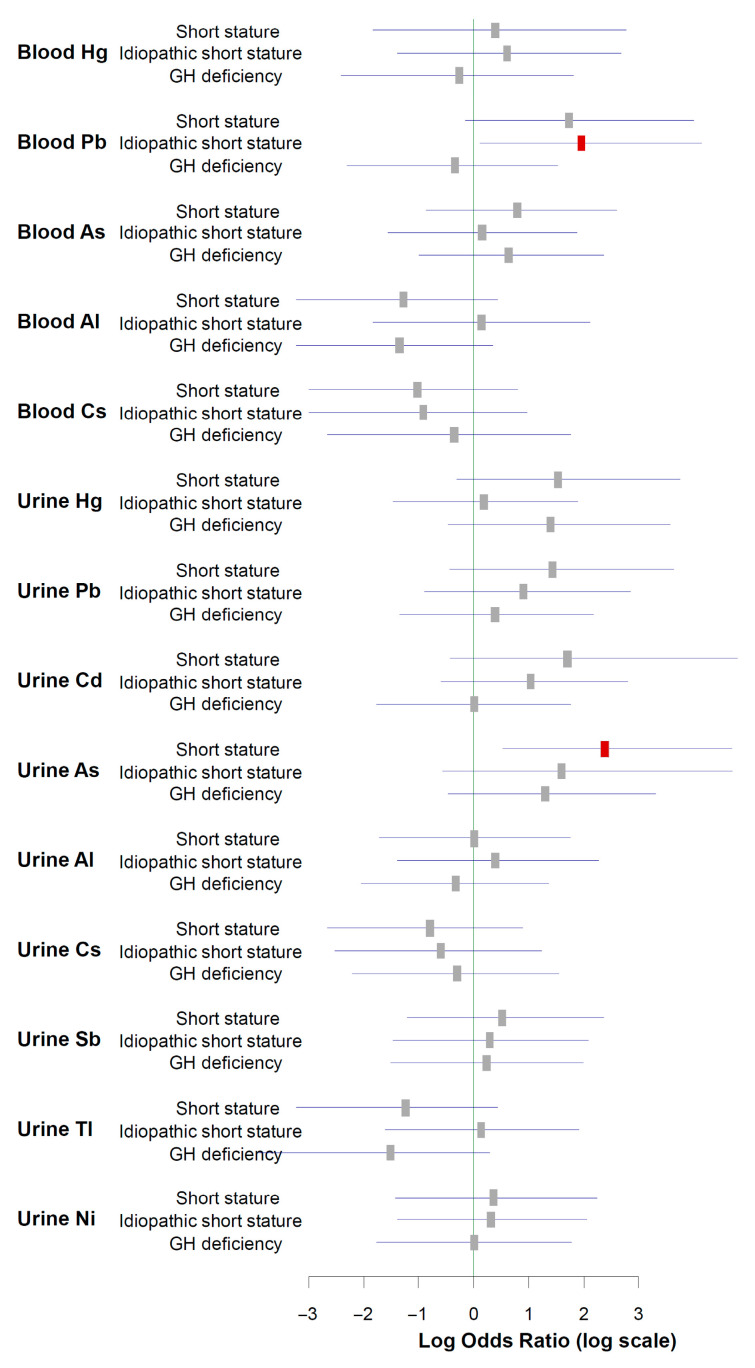
Tertile-based sensitivity analysis showing age- and sex-adjusted odds ratios (with 95% confidence intervals) for overall short stature, idiopathic short stature (ISS), and growth hormone deficiency (GHD), comparing the highest vs. lowest tertiles of blood and urinary heavy metal concentrations. Grey squares represent non-significant results, and red squares represent statistically significant results (*p* < 0.05).

To assess the robustness of the median-based findings, we performed a sensitivity analysis using tertile-based exposure cut-offs. Figure 1 presents age- and sex-adjusted odds ratios comparing the highest vs. lowest tertiles of heavy metal exposure. In these logistic regression models, children in the highest exposure tertile for blood Pb had higher odds of ISS, and those in the highest tertile for urinary As had higher odds of overall short stature than children in the lowest tertiles; both associations were statistically significant. For Hg, Cd, Ni, Tl, Sb, Al, and Cs, odds ratios did not show statistically significant associations with any short stature phenotype.

Quantile g-computation models were used to evaluate the joint effects of selected heavy metals on short stature phenotypes using tertile-based exposure categorization (q = 3) (Table 3). For overall short stature, a simultaneous one-quantile increase in the exposure mixture (blood Pb, urinary As, and urinary Hg) was associated with substantially higher odds (odds ratio OR 9.987; 95% confidence interval CI, 1.247–79.95). Within the mixture, urinary As carried the largest positive weight (0.466), followed by blood Pb (0.283) and urinary Hg (0.251).

For ISS, the mixture was also positively associated with increased odds (OR 3.691; 95% CI, 1.021–13.34), with contributions from blood Pb (weight 0.563) and urinary As (weight 0.437). For GHD, the mixture demonstrated a positive association with increased odds (OR 3.013; 95% CI, 1.728–5.252), with positive contributions from urinary As (weight 0.526) and urinary Hg (weight 0.474).

### 2.4. MiRNA Expression in Function of Metal Exposure and Short Stature Phenotype

**Figure 2:**
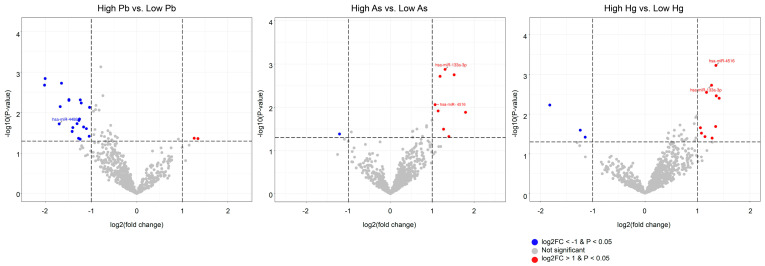
Volcano plots of differentially expressed miRNAs according to heavy metal exposure and short stature phenotype. Analyses are based on median-defined exposure categories (high vs. low). The *x*-axis shows log2 fold-change (positive values indicate upregulation and negative values indicate downregulation in the high-exposure group), and the *y*-axis shows −log10 *p*-values. Hsa-miR-4488 was downregulated in the high blood Pb exposure group and in ISS (vs. control). Hsa-miR-4516 and hsa-miR-133a-3p were upregulated in the high urinary Hg or As groups; hsa-miR-4516 was upregulated in GHD (vs. control and ISS), whereas hsa-miR-133a-3p was upregulated in GHD (vs. ISS).

Differential expression analysis revealed exposure- and phenotype-related miRNA signals (Figure 2; Tables S3 and S4). After Benjamini–Hochberg correction, none of the miRNAs reached the false discovery rate (FDR) < 0.05 threshold. Accordingly, miRNAs meeting the predefined criteria (raw *p* < 0.05 and |log2 fold-change (FC)| > 1) are presented as exploratory, hypothesis-generating signals. High blood Pb was associated with 22 miRNAs, mostly downregulated (20/22), including hsa-miR-4488 (log2FC = −1.704), which was also markedly reduced in ISS vs. control (log2FC = −2.437). High urinary Hg was associated with 13 miRNAs, largely upregulated (10/13), particularly hsa-miR-4516 (log2FC = 1.353) and hsa-miR-133a-3p (log2FC = 1.267). High urinary As was associated with nine miRNAs, mainly upregulated (8/9), including hsa-miR-133a-3p (log2FC = 1.306), and hsa-miR-4516 (log2FC = 1.069). Phenotype contrasts revealed 23 DEmiRNAs in ISS vs. control, 15 in GHD vs. control, and 19 in GHD vs. ISS. Recurrent signals included miR-4516 (upregulated with increased Hg/As levels and in GHD vs. both control and ISS) and miR-133a-3p (upregulated with increased As levels and in GHD vs. ISS), whereas miR-4488 was downregulated with increased Pb exposure and in ISS.

### 2.5. Predicted Targets of DEmiRNAs and Pathway Prioritization

Predicted targets for DEmiRNAs derived from high-vs.-low-exposure contrasts were identified from miRTarBase using Mienturnet (Tables S5–S7). We thus identified DEmiRNA target genes associated with growth-plate regulation, focusing on key pathways related to linear growth, including hypothalamic–pituitary–GH/IGF-1 axis signaling, chondrocyte proliferation, hypertrophy, and endochondral ossification (Table 4 and Table S8). In the high-Pb group, targets of hsa-miR-4488 (downregulated with high Pb and in ISS) included *RUNX3*, *SALL4*, *RXRB*, *MAPK8IP2*, *GNAI2*, *H2AFX*, and *THSD4.* Enrichr mapping indicated enrichment for TGF-β/BMP, WNT/β-catenin, NOTCH, and MAPK/JNK signaling and extracellular matrix-related processes. In the high-As group, hsa-miR-4516 and hsa-miR-133a-3p (both upregulated) targeted *ALDH9A1*, *KPNA6*, *NF2*, and *PTPN14*, with enrichment across Hippo/YAP, adhesion/FAK, PI3K-AKT/mTOR, nuclear import, and mitochondrial/stress pathways. In the high-Hg group, hsa-miR-4516 and hsa-miR-133a-3p targeted *BCL9L*, *STAT3*, *FOSL2*, *MC2R*, *ALDH9A1*, *RFT1*, *TRAF6*, and *KPNA6*, mapping to the WNT/β-catenin, JAK-STAT/cytokine, NF-κB/RANK, ACTH-cAMP, and N-glycosylation/ER processing pathways. These genes have been linked to short stature, growth-plate dysfunction, and impaired chondrocyte proliferation in previous human and animal studies (Table S9).

## 3. Discussion

### 3.1. Principal Findings

This clinical pilot study suggests a significant association between elevated exposure to Pb, As, and Hg and clinically diagnosed short stature in children. Elevated blood Pb levels were associated with ISS, whereas elevated urinary As levels were associated with short stature overall. Mixture modeling further suggested that blood Pb and urinary As were major contributors to ISS, whereas urinary As and Hg contributed to GHD within the exposure mixture. At the molecular level, hsa-miR-4488 was downregulated in both the high-Pb-exposure and ISS groups, whereas hsa-miR-133a-3p and hsa-miR-4516 were upregulated in children with increased urinary Hg or As levels and in those with GHD. Pathway analyses implicated these miRNAs in the regulation of genes involved in the GH/IGF-1 axis and endochondral ossification, suggesting that they may serve as mechanistic mediators and biomarkers of environmentally driven growth impairment.

### 3.2. Discussion in Light of Current Literature

Most previous epidemiological studies have reported modest associations between heavy metal exposure and childhood growth, typically small reductions in continuous height-related indices (approximately 0.1–0.3 SD per interquartile or tertile increase in exposure) in population-based cohorts [4,5,21]. In the present study, direct quantitative comparison of effect sizes with these previous reports is limited, as we did not analyze growth outcomes as continuous height SDS, but rather focused on clinically adjudicated short stature phenotypes. Nonetheless, we observed relatively large effect estimates in terms of odds ratios (approximately 3–10), particularly in mixture analyses. This difference likely reflects the use of dichotomous clinical outcomes—ISS and GHD—which focus on children at the extreme lower tail of the growth distribution, thereby concentrating susceptibility within a smaller and more homogeneous subgroup. Accordingly, the larger effect sizes observed in this study should be interpreted as a function of outcome definition and study design rather than as evidence of stronger causal effects, and our findings should be viewed as exploratory, warranting confirmation in larger and longitudinal studies.

The association between Pb exposure and impaired linear growth found in our study is consistent with a substantial body of evidence from diverse international cohorts. In a US cohort of young girls with a median blood Pb level of 9.9 µg/L, increased exposure was associated with reduced height in adolescence [5]. Similar associations with detrimental effects on linear growth and stunting risk have been documented in other cohorts with high-level exposures, including Mexican infants (median: 45 µg/L), Beninese toddlers (median: 59.3 µg/L), and Russian male adolescents (median: 30 µg/L) [22,23,24]. The median blood Pb concentration in our study cohort was 4.85 µg/L, which is notably lower than the levels reported in the above studies. This finding suggests that even relatively low-level Pb exposure, consistent with contemporary background levels in many regions, may adversely affect childhood growth, reinforcing the global relevance of Pb as a risk factor for growth impairment.

Similarly, the negative association between As exposure and linear growth observed in our study is supported by prior findings in diverse international cohorts. For example, in regions with high levels of environmental exposure, such as Bangladesh, elevated urinary As concentrations in children (medians ranging 51–80 µg/L) are inversely associated with height [12,25]. In Chinese children, urinary As concentrations (median: 40.75 µg/g creatinine) were negatively associated with height-for-age z-scores [26]. Importantly, this effect is not limited to high-exposure regions. A recent analysis of National Health and Nutrition Examination Survey data from US children, who had a relatively low median urinary As level of 6.4 µg/L, revealed that certain inorganic As metabolites were negatively associated with height z-scores, indicating that growth-related risks may occur even at lower exposure levels [4]. In our cohort, increased urinary As was a primary contributor to the risk of both overall short stature and GHD. The median urinary As concentration in our study participants was 84.36 µg/g creatinine, which is notably high and even exceeds the levels reported in studies in Bangladesh, where growth impacts were clearly observed. This elevated exposure is likely to reflect regional exposure characteristics. In Korea, dietary patterns are characterized by frequent consumption of seafood and rice, both recognized sources of As exposure. Seafood intake is primarily associated with organic As species, which are generally considered less toxic, whereas rice consumption may contribute to inorganic As exposure depending on cultivation and processing conditions [27]. Because detailed dietary intake data and As speciation were not available in the present study, we could not distinguish between organic and inorganic As or to quantitatively adjust for specific dietary sources. This limitation highlights the need for future studies incorporating repeated exposure measurements, dietary assessments, and As speciation to more precisely characterize the relationship between As exposure and growth-related outcomes.

Although most studies on Hg toxicity in children have focused on neurocognitive outcomes, emerging evidence indicates that Hg also exerts adverse effects on linear growth. In a longitudinal study in Poland, prenatal airborne Hg exposure was associated with slower height gain at the age of 9 years [28]. More direct evidence comes from a recent nationally representative study in Korea, which demonstrated that urinary Hg concentrations were inversely correlated with height z-scores in boys aged 3–5 years [14]. Moreover, urinary Hg levels in Korean children were two to three times higher than those reported in US, Canadian, and German cohorts, indicating substantial environmental exposure in the Korean population. Our observation of a borderline increase in the proportion of GHD with elevated urinary Hg levels is in line with previous studies suggesting a potential link between Hg exposure and impaired growth.

### 3.3. Biological Interpretation and Mechanisms

The altered circulating miRNAs identified in this study were implicated in growth-relevant biological pathways, particularly matrix remodeling, chondrocyte proliferation, and the GH/IGF-1 axis. Downregulation of hsa-miR-4488, which was significantly associated with both high-Pb exposure and ISS, was predicted to affect several genes central to skeletal growth. MicroRNAs canonically function as post-transcriptional repressors by binding to the 3′-UTR of target mRNAs, reducing protein translation or promoting mRNA degradation. Consequently, miR-4488 downregulation theoretically predicts increased expression of its target genes, particularly *RUNX3*, *SALL4*, *RXRB*, *MAPK8IP2*, *H2AFX*, *THSD4*, and *GNAI2*. These genes participate in endochondral ossification via core signaling pathways including TGF-β/BMP, WNT/β-catenin, NOTCH, and JNK/p38, and regulate extracellular matrix composition, which controls chondrocyte proliferation and hypertrophy [18]. Although we did not measure target protein levels, existing literature supports pathogenic roles for these genes in growth-plate biology and endocrine regulation. For instance, *RUNX3*, a Runt-domain transcription factor essential for chondrocyte maturation, plays a context-dependent role in endochondral ossification. *RUNX3* overexpression in proliferative-zone chondrocytes suppressed proliferation and accelerated hypertrophic differentiation, thereby shortening the proliferative zone and reducing linear growth velocity. MAPK8IP2, a scaffold protein that organizes JNK and p38 stress-activated kinase modules, may amplify cellular stress responses to Pb toxicity. JNK/p38 MAPK pathway activation promotes premature chondrocyte hypertrophy and suppresses proliferation, further compromising growth-plate function. Beyond local growth-plate effects, GNAI2 upregulation resulting from miR-4488 downregulation may contribute to the inhibition of pituitary GH secretion [29]. *GNAI2* encodes the Gαi2 subunit that transduces somatostatin receptor (SSTR2 and SSTR5) signaling in pituitary somatotrophs. Upon somatostatin binding, Gαi2 inhibits adenylyl cyclase, reducing cAMP production and PKA activity, which in turn suppresses GH secretion and counteracts GH-releasing hormone-stimulated GH release. Therefore, enhanced *GNAI2* expression may amplify endogenous somatostatin tone, leading to relative GH insufficiency even in the absence of overt GH deficiency. This interpretation is supported by our observation that children with ISS had significantly lower IGF-1 SDS values than controls (Table 1), suggesting impaired GH/IGF-1 axis function.

Upregulation of hsa-miR-4516 and hsa-miR-133a-3p was associated with increased urinary As and Hg concentrations in this study. These miRNAs had shared regulatory targets such as *KPNA6* and *ALDH9A1*, as well as metal-specific targets (i.e., *PTPN14* and *NF2* for As, and *STAT3*, *BCL9L*, *TRAF6*, *MC2R*, and *RFT1* for Hg). Many of these genes are key components of pathways central to endochondral ossification and growth-plate biology. Specifically, the As-responsive targets (*PTPN14* and *NF2*) function within the Hippo/YAP and adhesion/FAK signaling networks, which are crucial for chondrocyte proliferation and growth-plate architecture [30,31]. The Hg-associated targets *BCL9L* and *TRAF6* are involved in WNT pathway activation and NF-κB/RANK remodeling, processes required for bone turnover and growth-plate maturation [32,33]. *FOSL2* (Fra-2), another Hg-responsive target, encodes a component of the AP-1 transcription factor complex that promotes chondrocyte hypertrophic differentiation and osteoblast gene expression, thereby directly affecting the progression of endochondral ossification and growth-plate remodeling [34]. *ALDH9A1*, affected by both metals, participates in cellular metabolic and stress response pathways, including mitochondrial metabolism, aldehyde detoxification, and p53/NF-κB signaling, which influence growth-plate cell health [35].

Several gene targets of hsa-miR-4516 and hsa-miR-133a-3p are integral to the GH/IGF-1 axis. The shared target *KPNA6*, a nuclear-import adaptor essential for GH-driven skeletal development, represents a key mechanistic link [36]. *KPNA6* facilitates the nuclear import of transcription factors such as STAT3 (an Hg-linked target), a key component of the JAK2-STAT signaling pathway [37,38]. Therefore, the above two miRNAs may reduce the activities of *KPNA6* and *STAT3* and consequently perturb GH/IGF-1 signaling [37,38]. Dysregulation of *MC2R* (an Hg-linked target) may alter adrenal cortisol output. While excess glucocorticoids suppress the GH axis, normal cortisol levels are physiologically required for optimal GH action and IGF-1 production; therefore, disruptions in *MC2R* function may impair growth by disturbing this delicate balance [39]. Finally, *RFT1* (an Hg-linked target) is essential for the N-glycosylation required for the proper trafficking and function of GH and IGF-1 receptors [40].

Importantly, for selected miRNAs identified in this study, prior studies provide external experimental support for specific miRNA–target relationships. In particular, hsa-miR-133a-3p has been shown in independent experimental studies to regulate osteochondral lineage commitment and differentiation by directly targeting *RUNX2*, a master transcription factor essential for endochondral ossification and skeletal development, as demonstrated through functional assays in vitro models [41]. In addition, hsa-miR-133a has been reported to modulate the JAK/STAT signaling pathway, including *STAT3*-related signaling, through gain- and loss-of-function experiments in human cellular models [42]. Regulation of IGF1R by hsa-miR-133a-3p has also been reported in osteosarcoma cell models [43]. Together, these targets play central roles in chondrocyte proliferation and differentiation as well as GH/IGF-1 signaling, providing external biological support for the relevance of hsa-miR-133a-3p dysregulation observed in association with elevated As and Hg exposure and GHD in our cohort.

In contrast, for hsa-miR-4516 and hsa-miR-4488, direct experimental validation of targets involved in linear growth or growth-plate-specific regulation remains limited in the existing literature. Nevertheless, prior studies suggest that these miRNAs participate in growth-related signaling at the pathway level, including regulation of STAT3 signaling, cell-cycle control, extracellular matrix–integrin interactions, skeletal fragility, and broader metabolic and endocrine pathways [44,45,46,47]. Accordingly, interpretation of hsa-miR-4516 and hsa-miR-4488 in the present study relies on target prediction analyses combined with well-established gene–phenotype associations and should be regarded as hypothesis-generating.

### 3.4. Study Strengths and Limitations

A primary strength of this study is its integrative design, combining environmental exposure assessment, detailed molecular profiling, and rigorous clinical phenotyping. This multifaceted approach allowed for the exploration of potential biological mechanisms linking toxicants to clinical outcomes, moving beyond simple associations. Furthermore, the use of strictly defined clinical cases of ISS and GHD, confirmed through standardized endocrine testing, is a significant advantage as it provides a clearer and more clinically relevant signal than studies that rely on continuous anthropometric data from the general population.

Nonetheless, several limitations must be acknowledged. First, the modest sample size and imbalanced case–control ratio limit statistical power and preclude definitive causal inference; accordingly, this work should be regarded as exploratory and hypothesis-generating rather than confirmatory. Second, the cross-sectional design further limits causal interpretation, rendering the findings preliminary. In addition, residual confounding from unmeasured factors may exist. Although analyses were adjusted for age and sex, other variables such as concurrent environmental exposures or dietary factors (e.g., seafood or rice intake) were not fully captured. Finally, biomarker measurement error is possible, as single spot urine samples for metals may not accurately reflect long-term exposure.

## 4. Materials and Methods

### 4.1. Study Design and Participants

This single-center, cross-sectional study was conducted at the pediatric endocrinology clinic of Inje University Sanggye Paik Hospital. During the study period, all children who consecutively visited the clinic and met the initial screening criteria were evaluated for eligibility. The study was performed in accordance with the ethical standards established by the Institutional Review Board of Inje University Sanggye Paik Hospital, which approved the study protocol and all procedures involving human individuals (approval No. 2023-03-014). Informed consent was obtained from each participant prior to participation, and strict measures were implemented to protect the privacy and confidentiality of personal data throughout the study.

Children were classified as cases if they had short stature, defined as a height SDS < −2.0 for age and sex. All children with short stature underwent a standardized diagnostic workup, including detailed auxological assessment, two pharmacological GH stimulation tests (glucagon and arginine), and pituitary–hypothalamic magnetic resonance imaging to exclude structural abnormalities. GHD was defined based on auxology plus a subnormal peak GH level (<7 ng/mL in both tests) in accordance with the institutional protocol and assay-specific cut-offs. ISS was defined as height ≤ −2.0 SDS with normal GH secretion on both tests and no identifiable systemic, endocrine, nutritional, or chromosomal etiology on routine assessment. Throughout this manuscript, the term “short stature” refers exclusively to children diagnosed with GHD or ISS as defined above.

Exclusion criteria were predefined and applied prior to enrollment and included: small-for-gestational-age birth, familial short stature (either parent’s height SDS < −2.0, sex-specific), brain anomaly or tumor, known syndromic diagnosis or skeletal dysplasia, chronic systemic disease (renal, hepatic, gastrointestinal, or cardiac), known endocrine disorders other than GHD, prior or current recombinant GH therapy at the time of biospecimen collection, chronic glucocorticoid use, and acute intercurrent illness likely to affect growth or laboratory measurements. Children meeting any exclusion criteria were not enrolled in the study.

The control group was recruited from the same source population and consisted of hospital-based controls, defined as children visiting the same institution during the same study period for non–growth-related concerns. Controls were frequency-matched to cases by age and sex and were required to have normal stature (height between −1.5 and +1.5 SDS), no history of chronic disease, and no medications known to affect growth or endocrine function. Controls were not recruited from the general population but from the same clinical catchment area, ensuring comparability and minimizing potential selection bias.

### 4.2. Anthropometric Assessment

The height and weight of the participants (barefoot and in light clothing) were measured by trained staff using a wall-mounted stadiometer and a calibrated digital scale, respectively, and were recorded to the nearest 0.1 cm and 0.1 kg. Body mass index (BMI) was calculated as weight (kg) divided by height (m)^2^. Height and BMI z-scores (SDS) were derived using the lambda–mu–sigma method as described in the 2017 Korean National Growth Charts for children and adolescents [21].

### 4.3. Laboratory Analysis

#### 4.3.1. Biospecimen Collection and Processing

After an overnight fast, venous whole blood was drawn into trace element-certified tubes (metal-free, royal-blue top) and spot urine was collected in metal-free polypropylene containers. Samples were transported on cold packs to the analytical laboratory under chain-of-custody and processed within 24 h using trace-element clean techniques (acid-washed consumables, laminar-flow workspace). For miRNA profiling, serum was obtained in serum separator tubes, allowed to clot at room temperature, and centrifuged, and the supernatant was immediately aliquoted. Serum samples were stored at −80 °C, and freeze/thaw cycles were limited to one before analysis.

#### 4.3.2. Measurement of Serum IGF-1

Serum IGF-1 concentrations were measured using an electrochemiluminescence immunoassay (ECLIA) on a cobas e analyzer (Roche Diagnostics, Mannheim, Germany) with the Elecsys IGF-1 assay, following the manufacturer’s instructions; the assay’s analytical measuring range is approximately 3–1600 ng/mL, and intra- and inter-assay coefficients of variation in our laboratory were <10%. Age- and sex-specific IGF-1 SDS were calculated using the age- and sex-specific reference ranges provided by the assay manufacturer.

#### 4.3.3. Quantification of Blood and Urinary Metals and Metalloid Levels

Trace elements were measured using inductively coupled plasma-mass spectrometry. Blood and urine analyses were performed on Agilent 7900 and Agilent 7700 systems, respectively (Agilent Technologies, Tokyo, Japan). The target panel comprised Hg, Pb, As, Al, Cs, Cd, Sb, Tl, and Ni. Multi element calibration was performed using certified standards (IV ICPMS 71A/71B; Inorganic Ventures, Christiansburg, VA, USA) and single element standards (Sigma Aldrich, Saint-Louis, MO, USA). Matrix matched calibrators, procedural blanks, and internal standards were included in each batch to correct for drift and matrix effects. For blood samples, method performance based on laboratory validation yielded a limit of detection (LOD) of 0.03 µg/L and a limit of quantification (LOQ) of 0.10 µg/L for Cd, Hg, Pb, Al, and Cs; the LOQ for blood As was 0.09 µg/L. For urine samples, the LOQ was 0.1 µg/g creatinine for Hg, Pb, Cd, As, Al, Cs, Tl, and Ni, and 0.01 µg/g creatinine for Sb. Limits of detection for urinary metals were not routinely reported by the analytical laboratory, as LOQs represent the lowest concentrations with acceptable analytical accuracy and precision for clinical reporting; the corresponding LODs were lower than the reported LOQs.

Each analytical run included duplicate samples (≥10%), low/high-quality control (QC) materials, and spike recovery checks. For internal QC, ClinChek^®^ Whole Blood Control (Levels I–II) for blood and ClinChek^®^ Urine Control (Levels I–II) for urine (RECIPE Chemicals, München, Germany) were included in each run and at least every 50 samples. The laboratory has participated in multiple external proficiency schemes, including the College of American Pathologists proficiency testing program, Centers for Disease Control (CDC) and Prevention Lead and Multi-element Proficiency Program, CDC Environmental Quality Assurance Laboratory Program, German External Quality Assessment Scheme, and Quebec Multielement External Quality Assessment Scheme. Acceptance criteria followed trace element best practice (typical recoveries 85–115%; inter-assay coefficients of variation generally <10%). Laboratory personnel were blinded to case/control status, and case/control samples were interleaved across runs.

#### 4.3.4. Circulating Exosomal miRNA Sequencing

Serum aliquots were thawed on ice, and exosomal miRNAs were isolated and processed for small-RNA library construction using the QIAseq miRNA Library Kit (QIAGEN, Hilden, Germany) following an Ion Torrent–compatible workflow. Libraries were sequenced on an Ion Torrent platform using the Ion 550 Kit–Chef (Thermo Fisher–compatible workflow, Waltham, MA, USA). In brief, pre-adenylated 3′ and 5′ adapters were ligated to mature miRNAs, which were then reverse transcribed with a universal RT primer. Indexed libraries were amplified in a single unbiased polymerase chain reaction. Pooled libraries were sequenced on the Ion Torrent platform using the Ion 550 Kit–Chef (single-end, 87 bp reads). Base-calling and demultiplexing were performed in Torrent Suite v5.16.1. In total, 36 human serum exosomal RNA libraries were generated and sequenced (reference: GRCh38; miRBase v22.1). Raw reads were subjected to QC with FastQC v0.11.9 and adapter trimming with Cutadapt v2.8. Reads shorter than 18 nucleotides and low-quality reads were removed. Processed reads were aligned to reference miRNAs (miRBase v22.1) with Bowtie v1.1.1 and quantified. Putative novel miRNA discovery was performed using miRDeep2 v0.1.2.

### 4.4. Statistical Analysis

All analyses were performed in R (v4.2.2; R Foundation for Statistical Computing, Vienna, Austria) using two-sided tests with a significance threshold of α = 0.05. Analyses were pre-specified prior to data inspection. Continuous variables are summarized as mean ± SD or median (interquartile range) according to distributional characteristics; categorical variables are presented as counts (percentages). Distributions were assessed using Shapiro–Wilk testing. Group means were compared using Student’s *t*-test or one-way ANOVA (with Bonferroni post hoc tests) for approximately normal data and using Wilcoxon rank-sum/Kruskal–Wallis testing (with Dunn’s test and Bonferroni adjustment) otherwise. Categorical variables were analyzed using Fisher’s exact test. Urinary metals are reported as creatinine-adjusted concentrations (µg/g creatinine), blood metals are presented in µg/L. Values below the LOQ were imputed as LOQ/√2. Right-skewed distributions were log2-transformed for parametric analyses. For primary comparisons, each metal was dichotomized at the cohort median to define low vs. high exposure (blood Pb, urinary As, and urinary Hg). Tertiles were examined in sensitivity analyses for dose–response explorations.

Associations between exposure groups and clinical outcomes (overall short stature, ISS, and GHD) were estimated using multivariable logistic regression after adjustments for age and sex. To evaluate the joint effects of concurrent metals, we fitted quantile g-computation mixture models (qgcomp) to obtain the overall mixture OR per one-quantile increase and weights for each metal. Non-parametric bootstrap resampling (≥1000 iterations) provided standard errors and 95% CIs. Serum miRNA counts were normalized to reads per million (RPM) and analyzed on the log2(RPM + 1) scale. DEmiRNAs were identified using edgeR (v3.38.4), with the pre-specified design including both exposure (low vs. high median split for each metal) and diagnostic contrasts (normal vs. ISS, normal vs. GHD, GHD vs. ISS). Differential expression *p*-values were adjusted for multiple testing using the Benjamini–Hochberg false FDR procedure. When no miRNAs met the conventional FDR significance threshold in the primary analysis, miRNAs with raw *p* < 0.05 and |log2FC| > 1 were prioritized as exploratory candidates for hypothesis generation, consistent with recommended practices for exploratory miRNA and RNA-seq profiling studies in small-scale dataset [48]. 

Predicted targets of significant DEmiRNAs were inferred using Mienturnet (database: miRTarBase), restricting these to genes targeted by at least two miRNAs and *p* < 0.05. Pathway enrichment was assessed using Enrichr (KEGG 2021 Human; Reactome 2024), retaining pathways with adjusted *p* < 0.05. Biological prioritization was based on relevance to growth-plate biology and the hypothalamic–pituitary–GH axis.

## 5. Conclusions

This pilot study suggested an association between elevated Pb, As, and Hg exposure and clinically defined cases of ISS and GHD in children. Exploratory miRNA profiling identified consistent patterns, including downregulation of hsa-miR-4488 in Pb-associated ISS and upregulation of hsa-miR-4516 and hsa-miR-133a-3p in As/Hg-associated GHD, which may reflect potential biological pathways linking environmental metal exposure to impaired growth. Although these miRNA findings are hypothesis-generating, they highlight the possible relevance of environmental factors in childhood growth disorders and suggest that circulating miRNA signatures could serve as candidate biomarkers. Larger, longitudinal studies with independent validation are needed to confirm these observations and to clarify the long-term impact of heavy metal exposure on pediatric growth outcomes.

## Figures and Tables

**Table 1:** General characteristics of study participants.

Variables	ISS (*n* = 13)	GHD (*n* = 11)	Control (*n* = 12)	*p*-Value
Male (%)	6 (46.2%)	4 (36.4%)	5 (41.7%)	0.915
CA (y)	6.56 (6.25, 8.67)	8.25 (5.75, 9.66)	7.33 (6.33, 8.56)	0.830
BA (y)	5.50 (5.00, 6.75)	6.50 (3.88, 7.44)	7.75 (6.50, 9.12)	0.091
CA − BA (y)	1.00 (0.92, 1.31)	1.50 (1.00, 2.52)	−0.21 (−1.29, 0.81)	0.003 ^‡^
Height (cm)	108.7 (107.0, 120.0)	115.7 (105.2, 125.6)	127.4 (119.2, 133.6)	0.009 ^†^
Weight (kg)	18.9 (17.4, 20.5)	17.7 (16.4, 24.4)	27.1 (24.2, 34.7)	0.002 ^†,‡^
BMI (kg/m^2^)	15.14 (14.98, 15.99)	15.12 (14.04, 16.89)	17.24 (15.64, 19.07)	0.029 ^†^
IGF-1 (ng/mL)	107.0 (88.2–123.0)	89.9 (78.8–120.5)	177.5 (154.5–229.0)	<0.001 ^†,‡^
Height SDS	−2.10 (−2.33, −1.94)	−2.00 (−2.21, −1.95)	0.25 (−0.23, 0.90)	<0.001 ^†,‡^
Weight SDS	−1.65 (−2.07, −1.21)	−1.72 (−2.44, −1.14)	0.01 (−0.42, 1.64)	<0.001 ^†,‡^
BMI SDS	−0.57 (−1.30, −0.09)	−0.51 (−1.75, 0.05)	−0.04 (−0.39, 1.66)	0.063
IGF-1 SDS	−1.21 (−1.30, −0.68)	−1.57 (−1.64, −0.84)	−0.34 (−0.71, 0.22)	<0.001 ^†,‡^

Values are median (interquartile range) or n (%). *p*-values: Kruskal–Wallis with Dunn’s test (Bonferroni) for continuous variables; Fisher’s exact for categorical variables. CA − BA was calculated as chronological age minus bone age; negative values indicate advanced bone age, whereas positive values indicate delayed bone age. ^†^ *p* < 0.05 ISS vs. control. ^‡^ *p* < 0.05 GHD vs. control. CA, chronological age; BA, bone age; BMI, body mass index; IGF-1, insulin-like growth factor-1; ISS, idiopathic short stature; GHD, idiopathic growth hormone deficiency; SDS, standard deviation score.

**Table 2:** Comparison of short stature proportion (*n*, %) by blood or urinary heavy metal concentrations (below median vs. at or above median) in study participants.

	Short Stature			ISS			GHD		
	Heavy Metal		*p*-Value	Heavy Metal		*p*-Value	Heavy Metal		*p*-Value
	Low	High		Low	High		Low	High	
Blood									
Hg	12 (66.7%)	12 (66.7%)	0.999	7 (38.9%)	6 (33.3%)	0.728	5 (27.8%)	6 (33.3%)	0.717
Pb	9 (50.0%)	15 (83.3%)	0.034	3 (16.7%)	10 (55.6%)	0.015	6 (33.3%)	5 (27.8%)	0.717
As	10 (55.6%)	14 (77.8%)	0.289	5 (27.8%)	8 (44.4%)	0.298	5 (27.8%)	6 (33.3%)	0.717
Al	13 (72.2%)	11 (61.1%)	0.480	6 (33.3%)	7 (38.9%)	0.729	7 (38.9%)	4 (22.2%)	0.278
Cs	14 (77.8%)	10 (55.6%)	0.157	7 (38.9%)	6 (33.3%)	0.729	7 (38.9%)	4 (22.2%)	0.278
Urine									
Hg	10 (55.6%)	14 (77.8%)	0.157	7 (38.9%)	6 (33.3%)	0.729	3 (16.7%)	8 (44.4%)	0.070
Pb	11 (61.1%)	13 (72.2%)	0.724	6 (33.3%)	7 (38.9%)	0.729	5 (27.8%)	6 (33.3%)	0.717
Cd	11 (57.9%)	13 (76.5%)	0.238	5 (26.3%)	8 (47.1%)	0.196	6 (31.6%)	5 (29.4%)	0.888
As	9 (50.0%)	15 (83.3%)	0.034	5 (27.8%)	8 (44.4%)	0.298	4 (22.2%)	7 (38.9%)	0.278
Al	12 (66.7%)	12 (66.7%)	0.999	5 (27.8%)	8 (44.4%)	0.298	7 (38.9%)	4 (22.2%)	0.278
Cs	14 (73.7%)	10 (58.8%)	0.345	8 (42.1%)	5 (29.4%)	0.429	6 (31.6%)	5 (29.4%)	0.888
Sb	13 (65.0%)	11 (68.8%)	0.999	6 (30.0%)	7 (43.8%)	0.393	7 (35.0%)	4 (25.0%)	0.517
Tl	13 (72.2%)	11 (61.1%)	0.480	7 (38.9%)	6 (33.3%)	0.729	6 (33.3%)	5 (27.8%)	0.717
Ni	10 (55.6%)	14 (77.8%)	0.157	5 (27.8%)	8 (44.4%)	0.298	5 (27.8%)	6 (33.3%)	0.717

Values are presented as *n* (%). Short stature comprises two mutually exclusive diagnostic categories: ISS and GHD. For each metal, exposure categories were defined by a median split (low: <median; high: ≥median) within the total study population. Percentages represent the proportion of children with each outcome (overall short stature, ISS, or GHD) within each exposure category.

**Table 3:** Relative contribution of individual metals and combined effects of metal mixtures on the likelihood of overall short stature, ISS, and GHD estimated using tertile-based quantile g-computation models (q = 3).

	Relative Weights			OR (95% CI)	
	Blood Pb	Urine As	Urine Hg		
Short stature	0.283	0.466	0.251	9.987	(1.247, 79.95)
ISS	0.563	0.437	0	3.691	(1.021, 13.34)
GHD	0	0.526	0.474	3.013	(1.728, 5.252)

Adjusted for sex and age. OR, odds ratio; CI, confidence intervals.

**Table 4:** Proposed mechanistic pathways linking heavy metal exposure to growth impairment via dysregulation of sentinel miRNAs and their gene targets.

Exposure Signal	Sentinel miRNA(s)	Predicted Targets	Key Pathways of Linear Growth	
			Enchondral Ossification	GH–IGF-1 Axis
High blood Pb	↓ miR-4488	*RUNX3*, *SALL4*, *RXRB*, *MAPK8IP2*, *THSD4*, *H2AFX*, *GNAI2*	Yes—TGF-β/BMP, WNT/β-catenin, NOTCH, JNK/p38; ECM microfibrils (*THSD4*) regulating chondrocyte proliferation and hypertrophy	Adjunct—*GNAI2* links to somatostatin (SSTR2/5, Gi/o) signaling that suppresses pituitary GH release
High urinary As	↑ miR-4516, ↑ miR-133a-3p	*KPNA6*, *PTPN14*, *NF2*, *ALDH9A1*	Yes—Hippo/YAP and adhesion/FAK programs modulate proliferative and hypertrophic zones (*PTPN14*, *NF2*); metabolic stress nodes (*ALDH9A1*)	Yes (supporting)—*KPNA6* (nuclear import) can modulate GH-responsive transcription
High urinary Hg	↑ miR-4516, ↑ miR-133a-3p	*STAT3*, *BCL9L*, *FOSL2*, *TRAF6*, *MC2R*, *RFT1*, *KPNA6*, *ALDH9A1*	Yes—WNT co-activation (*BCL9L*), NF-κB/RANK remodeling (*TRAF6*), and AP-1 complex activation (*FOSL2*) influence plate maturation and bone turnover; metabolic stress nodes (*ALDH9A1*)	Yes (adjacent)—↓*MC2R*: decreases cortisol (permissive for GH), which may lower GH pulse amplitude and hepatic GH–STAT5 signaling, reducing IGF-1; *STAT3*: intersects inflammatory GH resistance; *RFT1*: N-glycosylation/trafficking of GHR/IGF1R; *KPNA6* (nuclear import) can modulate GH-responsive transcription

The upward arrow (↑) indicates upregulation, and the downward arrow (↓) indicates downregulation.

## Data Availability

The data presented in this study are available on request from the corresponding authors due to privacy and ethical restrictions concerning human study participants.

## Supplementary Materials

The following supporting information can be downloaded at: https://www.mdpi.com/article/10.3390/ijms27031230/s1. References [49,50,51,52,53,54,55,56,57,58,59,60,61,62,63,64,65,66,67,68,69,70,71,72,73,74,75,76] are cited in the Supplementary Material.

## Abbreviations

The following abbreviations are used in this manuscript:

BMI	Body mass index
CDC	Centers for Disease Control
CI	Confidence interval
CV	Coefficient of variation
DEmiRNA	Differentially expressed miRNA
FDR	False discovery rate
FC	Fold-change
GHD	Growth hormone deficiency
GH	Growth hormone
IGF-1	Insulin-like growth factor-1
ISS	Idiopathic short stature
LOD	Limit of detection
LOQ	Limit of quantification
OR	Odds ratio
QC	Quality control
RPM	Reads per million
SDS	Standard deviation score
