# Supplementary material for: Circulating Exosomal MicroRNA Profiles Associated with Heavy Metal Exposure and Short Stature in Children
Source: Int J Mol Sci. 2026 Jan 26;27(3):1230. doi: 10.3390/ijms27031230 (PMC12898619; doi:10.3390/ijms27031230)
Supplement: Supplementary file 1 [file ijms-27-01230-s001.zip › ijms-4062441-supplementary.pdf]

## Supplementary Material

**Figure S1.** Log-transformed distributions of blood and urinary metal concentrations in study participants.

**Figure S2.** Correlation heatmap of heavy metal and metalloid concentrations in the blood and urine. Only statistically significant correlation coefficients are presented.

**Table S1.** Distribution of blood and urine concentrations of heavy metal and metalloid in study participants.

**Table S2.** Comparison of blood and urine concentrations of heavy metal and metalloid in study participants.

**Table S3.** Differentially expressed miRNAs ($|log2FC| > 1$, $p < 0.05$) between the high and low exposure groups for blood Pb, urine Hg, and urine As.

**Table S4.** Differentially expressed miRNAs ($|log2FC| > 1$, $p < 0.05$) according to the height groups.

**Table S5.** Predicted target genes of differentially expressed miRNAs in the high blood Pb group compared with the low blood Pb group.

**Table S6.** Predicted target genes of differentially expressed miRNAs in the high urinary As group compared with the low As group.

**Table S7.** Predicted target genes of differentially expressed miRNAs in the high urinary Hg group compared with the low Hg group.

**Table S8.** Predicted target genes of differentially expressed miRNAs in the high heavy metal exposure group associated with growth plate regulation, focusing on key pathways of linear growth, including hypothalamic–pituitary–GH/IGF-1 axis signaling, chondrocyte proliferation, hypertrophy, and endochondral ossification.

**Table S9.** Reported growth-related phenotypes of predicted target genes regulated by differentially expressed miRNAs in the high heavy metal exposure group.

**Figure S1.** Log-transformed distributions of blood and urinary metal concentrations in study participants.

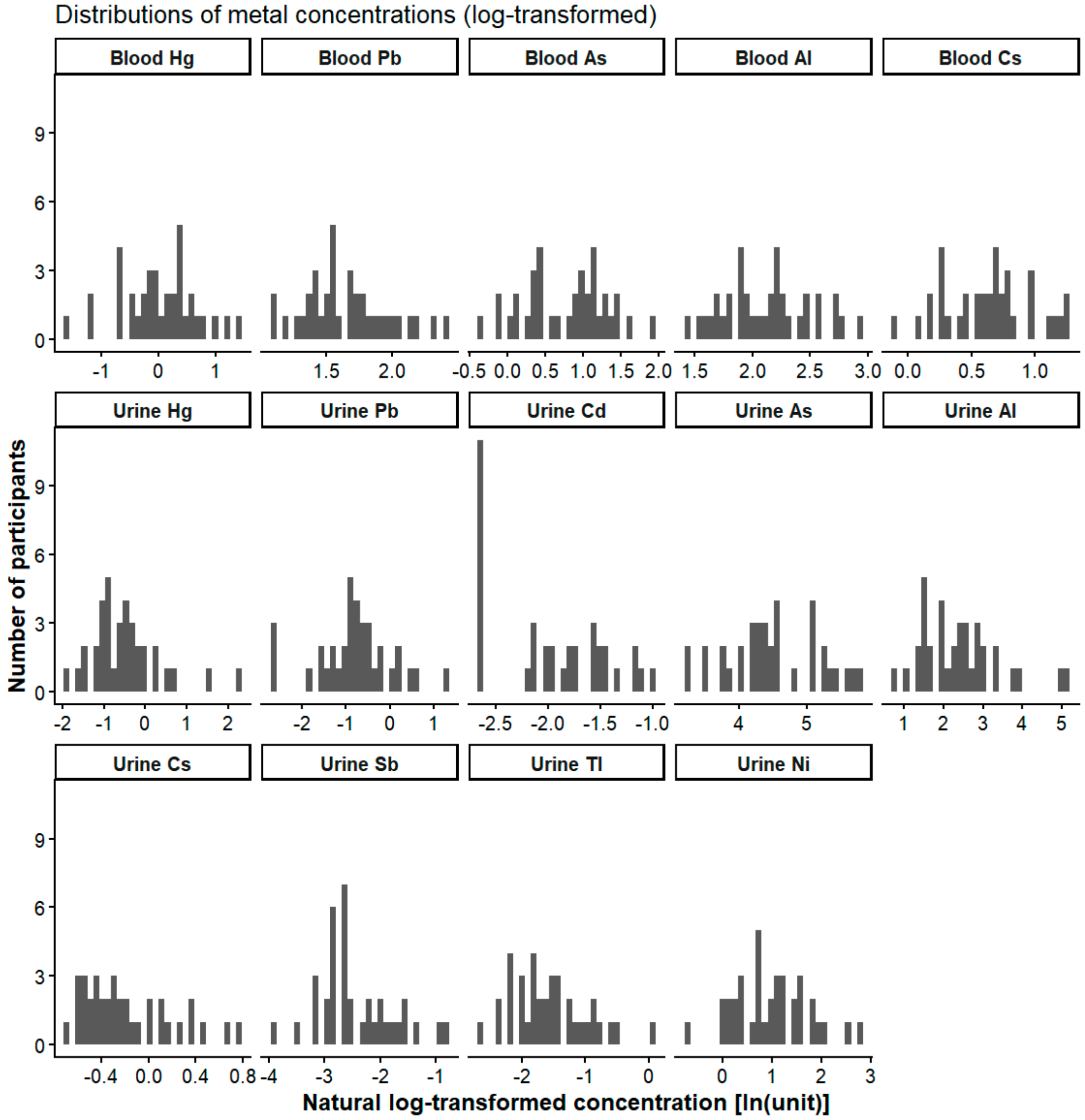

**Figure S2.** Correlation heatmap of heavy metal and metalloid concentrations in the blood and urine. Only statistically significant correlation coefficients are presented.

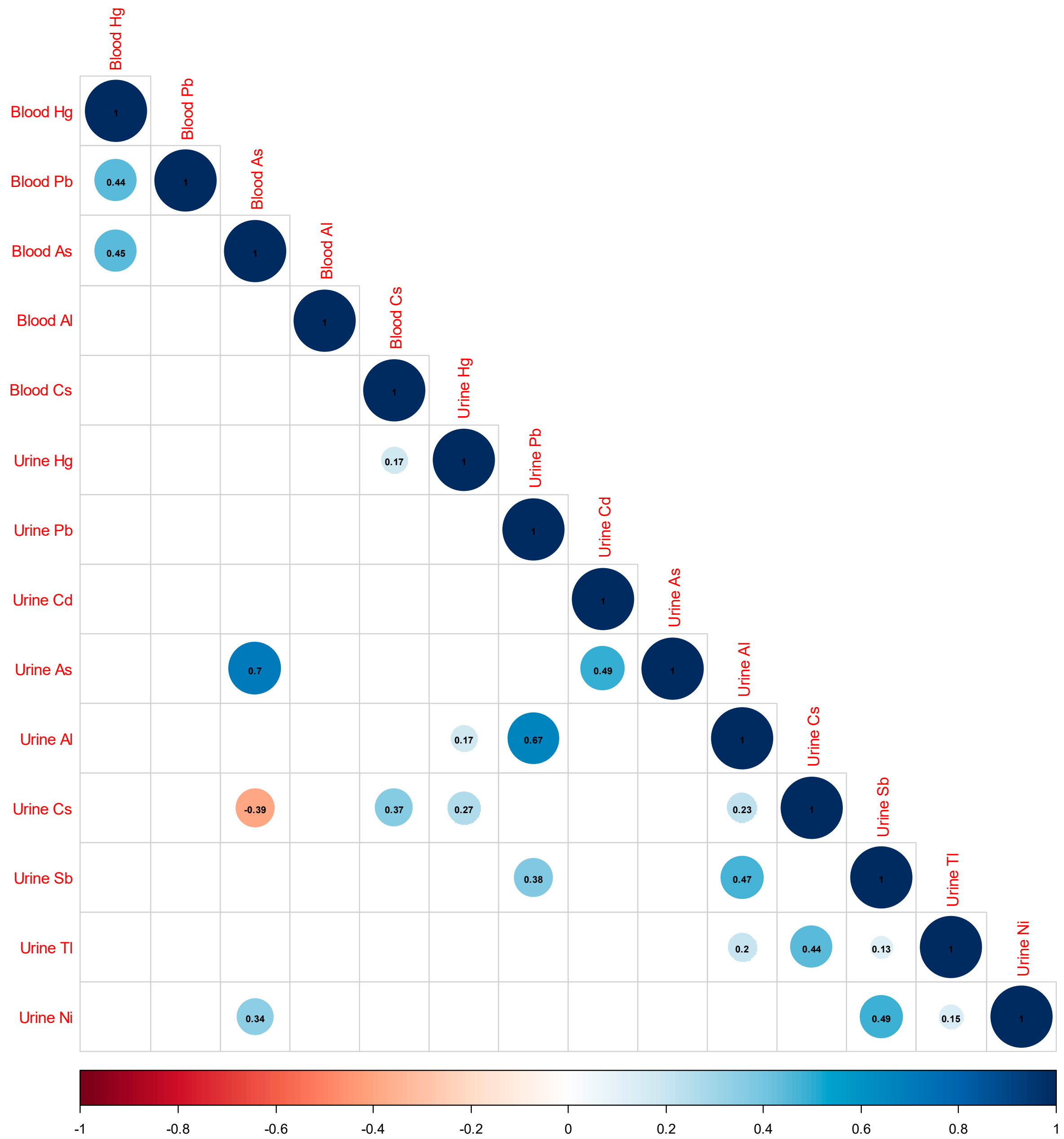

**Table S1.** Distribution of blood and urine concentrations of heavy metal and metalloid in study participants.

| | LOQ | Percentage above LOQ | Median (interquartile range) | Selected percentiles | | |
|---|---|---|---|---|---|---|
| | | | | 33th | 50th | 66th |
| Blood (μg/L) | | | | | | |
| Blood Hg | 0.1 | 100.0 | 1.05 (0.67, 1.42) | 0.86 | 1.05 | 1.40 |
| Blood Pb | 0.1 | 100.0 | 4.85 (4.18, 6.3) | 4.60 | 4.85 | 5.73 |
| Blood As | 0.09 | 100.0 | 2.45 (1.4, 3.2) | 1.60 | 2.45 | 2.92 |
| Blood Al | 0.1 | 100.0 | 8.4 (6.45, 10.35) | 6.80 | 8.4 | 9.32 |
| Blood Cs | 0.1 | 100.0 | 1.95 (1.48, 2.22) | 1.66 | 1.95 | 2.11 |
| Urine (μg/g Creatinine) | | | | | | |
| Urine Hg | 0.1 | 100.0 | 0.56 (0.38, 0.86) | 0.41 | 0.56 | 0.69 |
| Urine Pb | 0.1 | 91.7 | 0.45 (0.32, 0.63) | 0.38 | 0.45 | 0.57 |
| Urine Cd | 0.1 | 69.4 | 0.14 (0.07, 0.21) | 0.12 | 0.14 | 0.18 |
| Urine As | 0.1 | 100.0 | 84.36 (62.91, 163.09) | 69.4 | 84.36 | 100.03 |
| Urine Al | 0.1 | 100.0 | 9.91 (5.19, 18.15) | 6.90 | 9.91 | 13.67 |
| Urine Cs | 0.1 | 100.0 | 0.76 (0.63, 1.02) | 0.65 | 0.76 | 0.86 |
| Urine Sb | 0.01 | 100.0 | 0.07 (0.06, 0.13) | 0.06 | 0.07 | 0.11 |
| Urine Tl | 0.1 | 100.0 | 0.20 (0.14, 0.29) | 0.16 | 0.20 | 0.23 |
| Urine Ni | 0.1 | 100.0 | 2.51 (1.51, 4.34) | 2.00 | 2.51 | 3.28 |

LOQ, limit of quantification

**Table S2.** Comparison of blood and urine concentrations of heavy metal and metalloid in study participants

| Variables | ISS (n = 13) | GHD (n = 11) | Control (n = 12) | p-value | ISS vs. control | GHD vs. control | ISS vs. GHD |
|---|---|---|---|---|---|---|---|
| **Blood (μg/L)** | | | | | | | |
| Hg | 1.00 (0.80, 1.80) | 1.10 (0.50, 1.50) | 1.10 (0.83, 1.40) | 0.727 | NS | NS | NS |
| Pb | 6.20 (5.30, 7.50) | 4.80 (4.2, 5.85) | 4.65 (4.10, 4.82) | 0.123 | NS | NS | NS |
| As | 2.60 (1.60, 3.10) | 2.60 (1.55, 3.50) | 1.70 (1.08, 2.83) | 0.293 | NS | NS | NS |
| Al | 8.80 (6.90, 9.30) | 6.00 (5.30, 9.85) | 9.55 (7.42, 13.62) | 0.176 | NS | NS | NS |
| Cs | 1.80 (1.40, 2.00) | 1.90 (1.50, 2.05) | 2.40 (1.60, 2.78) | 0.217 | NS | NS | NS |
| **Urine (μg/g creatinine)** | | | | | | | |
| Hg | 0.42 [0.38, 0.9] | 0.63 [0.57, 0.81] | 0.45 [0.40, 0.60] | 0.557 | NS | NS | NS |
| Pb | 0.47 [0.41, 0.62] | 0.48 [0.18, 0.72] | 0.40 [0.34, 0.55] | 0.753 | NS | NS | NS |
| Cd | 0.21 [0.08, 0.22] | 0.14 [0.10, 0.29] | 0.12 [0.11, 0.16] | 0.383 | NS | NS | NS |
| As | 91.69 [82.97, 165.37] | 162.41 [64.81, 188.56] | 65.96 [39.06, 78.89] | 0.023 | 0.038 | 0.046 | 0.904 |
| Al | 11.15 [7.31, 20.42] | 8.22 [4.85, 18.01] | 9.50 [5.105, 15.68] | 0.747 | NS | NS | NS |
| Cs | 0.72 [0.61, 0.82] | 0.76 [0.62, 0.88] | 0.89 [0.64, 1.44] | 0.335 | NS | NS | NS |
| Sb | 0.08 [0.06, 0.12] | 0.07 [0.06, 0.16] | 0.07 [0.05, 0.11] | 0.697 | NS | NS | NS |
| Tl | 0.19 [0.14, 0.28] | 0.16 [0.13, 0.23] | 0.23 [0.17, 0.41] | 0.252 | NS | NS | NS |
| Ni | 3.08 [1.98, 4.68] | 2.53 [1.53, 4.08] | 2.16 [1.45, 3.51] | 0.648 | NS | NS | NS |

Results are presented as median (interquartile range) and comparisons were performed using the Kruskal–Wallis test with Dunn's test for post-hoc analysis.

ISS, idiopathic short stature; GHD, idiopathic growth hormone deficiency

**Table S3.** Differentially expressed miRNAs (|log2FC| > 1, p < 0.05) between the high and low exposure groups for blood Pb, urine Hg, and urine As.

| **High vs. low blood Pb** | | | | **High vs. low urine As** | | | | **High vs. low urine Hg** | | | |
|---|---|---|---|---|---|---|---|---|---|---|---|
| **Mature miRNA** | **Log2FC** | **p-value** | **FDR** | **Mature miRNA** | **Log2FC** | **p-value** | **FDR** | **Mature miRNA** | **Log2FC** | **p-value** | **FDR** |
| hsa-miR-4755-3p | 1.342 | 0.043 | 0.746 | hsa-miR-4488 | 1.795 | 0.013 | 0.999 | hsa-miR-3614-5p | 1.415 | 0.004 | 0.413 |
| hsa-miR-378g | 1.261 | 0.043 | 0.746 | hsa-miR-3614-5p | 1.523 | 0.002 | 0.447 | hsa-miR-6724-5p | 1.359 | 0.003 | 0.405 |
| hsa-miR-766-5p | −1.037 | 0.007 | 0.563 | hsa-miR-6821-5p | 1.397 | 0.047 | 0.999 | hsa-miR-4516† | 1.353 | 0.001 | 0.405 |
| hsa-miR-6772-3p | −1.045 | 0.038 | 0.746 | hsa-miR-133a-3p‡ | 1.306 | 0.001 | 0.447 | hsa-miR-376b-3p | 1.348 | 0.020 | 0.910 |
| hsa-miR-511-5p | −1.106 | 0.025 | 0.746 | hsa-miR-3183 | 1.270 | 0.032 | 0.999 | hsa-miR-3180-3p | 1.280 | 0.040 | 0.910 |
| hsa-miR-6837-3p | −1.167 | 0.022 | 0.746 | hsa-miR-1246 | 1.187 | 0.002 | 0.447 | hsa-miR-133a-3p‡ | 1.267 | 0.002 | 0.405 |
| hsa-miR-12136 | −1.219 | 0.006 | 0.563 | hsa-miR-4676-3p | 1.141 | 0.012 | 0.999 | hsa-miR-450a-1-3p | 1.173 | 0.003 | 0.405 |
| hsa-miR-4448 | −1.240 | 0.005 | 0.563 | hsa-miR-4516† | 1.069 | 0.009 | 0.999 | hsa-miR-1234-3p | 1.144 | 0.037 | 0.910 |
| hsa-miR-3180-3p | −1.243 | 0.045 | 0.746 | hsa-miR-548j-3p | −1.214 | 0.041 | 0.999 | hsa-miR-6807-5p | 1.077 | 0.030 | 0.910 |
| hsa-miR-4748 | −1.257 | 0.014 | 0.746 | | | | | hsa-miR-2115-3p | 1.057 | 0.022 | 0.910 |
| hsa-miR-365b-5p | −1.269 | 0.015 | 0.746 | | | | | hsa-miR-3192-5p | −1.143 | 0.038 | 0.910 |
| hsa-miR-4446-5p | −1.278 | 0.043 | 0.746 | | | | | hsa-miR-34b-3p | −1.237 | 0.025 | 0.910 |
| hsa-miR-3942-5p | −1.311 | 0.019 | 0.746 | | | | | hsa-miR-6515-5p | −1.819 | 0.006 | 0.556 |
| hsa-let-7e-3p | −1.404 | 0.023 | 0.746 | | | | | | | | |
| hsa-miR-4773 | −1.419 | 0.029 | 0.746 | | | | | | | | |
| hsa-miR-2116-5p | −1.489 | 0.005 | 0.746 | | | | | | | | |
| hsa-miR-1185-2-3p | −1.489 | 0.005 | 0.746 | | | | | | | | |
| hsa-miR-4725-3p | −1.653 | 0.002 | 0.746 | | | | | | | | |
| hsa-miR-181d-3p | −1.681 | 0.007 | 0.746 | | | | | | | | |
| hsa-miR-4488* | −1.704 | 0.019 | 0.746 | | | | | | | | |
| hsa-miR-4632-3p | −2.011 | 0.001 | 0.746 | | | | | | | | |
| hsa-miR-508-3p | −2.025 | 0.002 | 0.746 | | | | | | | | |

*Also downregulated in the ISS group compared with the control group

†Also upregulated in the GHD group compared with the control and ISS groups

‡Also upregulated in the GHD group compared with the ISS group

**Table S4.** Differentially expressed miRNAs (|log2FC| > 1, p < 0.05) according to the height groups.

| ISS vs. control | | | | GHD vs. control | | | | GHD vs. ISS | | | |
|---|---|---|---|---|---|---|---|---|---|---|---|
| **Mature miRNA** | **Log2FC** | **p-value** | **FDR** | **Mature miRNA** | **Log2FC** | **p-value** | **FDR** | **Mature miRNA** | **Log2FC** | **p-value** | **FDR** |
| hsa-miR-6515-5p | 1.804 | 0.040 | 0.813 | hsa-miR-6802-3p | 1.768 | 0.010 | 0.978 | hsa-miR-4687-5p | 2.059 | 0.005 | 0.999 |
| hsa-miR-409-5p | 1.801 | 0.008 | 0.761 | hsa-miR-409-5p | 1.642 | 0.026 | 0.978 | hsa-miR-3190-3p | 1.783 | 0.008 | 0.999 |
| hsa-miR-12135 | 1.691 | 0.019 | 0.813 | hsa-miR-543 | 1.494 | 0.020 | 0.978 | hsa-miR-19b-1-5p | 1.768 | 0.022 | 0.999 |
| hsa-miR-2278 | 1.558 | 0.010 | 0.761 | hsa-miR-627-5p | 1.412 | 0.003 | 0.896 | hsa-miR-1236-5p | 1.688 | 0.022 | 0.999 |
| hsa-miR-6882-5p | 1.513 | 0.018 | 0.813 | hsa-miR-3190-3p | 1.386 | 0.018 | 0.978 | hsa-miR-1185-2-3p | 1.672 | 0.02 | 0.999 |
| hsa-miR-323b-3p | 1.450 | 0.013 | 0.761 | hsa-miR-3191-3p | 1.347 | 0.033 | 0.978 | hsa-miR-6802-3p | 1.634 | 0.012 | 0.999 |
| hsa-miR-1262 | 1.348 | 0.049 | 0.813 | hsa-miR-6741-3p | 1.165 | 0.002 | 0.896 | hsa-miR-3613-3p | 1.567 | 0.043 | 0.999 |
| hsa-miR-4646-5p | 1.242 | 0.018 | 0.813 | hsa-miR-4516† | 1.132 | 0.045 | 0.978 | hsa-miR-873-3p | 1.472 | 0.038 | 0.999 |
| hsa-miR-4433b-3p | 1.223 | 0.012 | 0.761 | hsa-miR-9-3p | 1.070 | 0.032 | 0.978 | hsa-miR-133a-3p‡ | 1.402 | 0.002 | 0.869 |
| hsa-miR-6813-5p | 1.218 | 0.022 | 0.813 | hsa-miR-92b-5p | 1.066 | 0.008 | 0.978 | hsa-miR-1275 | 1.347 | 0.027 | 0.999 |
| hsa-miR-548av-5p | 1.126 | 0.047 | 0.813 | hsa-miR-5193 | 1.058 | 0.015 | 0.978 | hsa-miR-1-3p | 1.162 | 0.009 | 0.999 |
| hsa-miR-548k | 1.126 | 0.047 | 0.813 | hsa-miR-6513-5p | 1.022 | 0.026 | 0.978 | hsa-miR-874-5p | 1.126 | 0.013 | 0.999 |
| hsa-miR-331-5p | 1.114 | 0.006 | 0.761 | hsa-miR-99b-3p | 1.021 | 0.016 | 0.978 | hsa-miR-1-3p | 1.118 | 0.012 | 0.999 |
| hsa-miR-3679-5p | 1.086 | 0.048 | 0.813 | hsa-miR-4804-5p | −1.131 | 0.024 | 0.978 | hsa-miR-4516† | 1.091 | 0.039 | 0.999 |
| hsa-miR-27a-5p | 1.072 | 0.030 | 0.813 | hsa-miR-365a-5p | −1.269 | 0.036 | 0.978 | hsa-miR-4665-5p | 1.035 | 0.045 | 0.999 |
| hsa-miR-23a-5p | 1.071 | 0.012 | 0.761 | | | | | hsa-miR-6859-5p | 1.033 | 0.025 | 0.999 |
| hsa-miR-330-3p | 1.054 | 0.026 | 0.813 | | | | | hsa-miR-1234-3p | −1.384 | 0.049 | 0.999 |
| hsa-miR-548e-5p | 1.036 | 0.023 | 0.813 | | | | | hsa-miR-3667-5p | −1.462 | 0.031 | 0.999 |
| hsa-miR-4526 | −1.040 | 0.037 | 0.813 | | | | | hsa-miR-4804-5p | −1.676 | 0.009 | 0.999 |
| hsa-miR-150-5p | −1.215 | 0.000 | 0.134 | | | | | | | | |
| hsa-miR-6724-5p | −1.227 | 0.034 | 0.813 | | | | | | | | |
| hsa-miR-1289 | −1.502 | 0.010 | 0.761 | | | | | | | | |
| hsa-miR-4488* | −2.437 | 0.007 | 0.761 | | | | | | | | |

Raw p-values were obtained from the edgeR likelihood ratio tests.

*Also downregulated in high blood Pb group compared with the low urine Pb group

†Also upregulated in the high urine Hg and As groups compared with the low groups

‡Also upregulated in the high urine As group compared with the low urine As group

ISS, idiopathic short stature; GHD, idiopathic growth hormone deficiency.

**Table S5.** Predicted target genes of differentially expressed miRNAs in the high blood Pb group compared with the low blood Pb group.

| Gene symbol | p-value | Odd ratio | Number of interactions | microRNA 1 | microRNA 2 | microRNA 3 | microRNA 4 | microRNA 5 | microRNA 6 |
|---|---|---|---|---|---|---|---|---|---|
| ***FXYD1*** | 0.0000464 | 0.0539 | 4 | **hsa-miR-4488** | hsa-miR-3180-3p | hsa-miR-3180 | hsa-miR-4725-3p | | |
| ***WSCD1*** | 0.0000619 | 0.0282 | 3 | **hsa-miR-4488** | hsa-miR-3180-3p | hsa-miR-3180 | | | |
| *GLIPR1L2* | 0.000248 | 0.0436 | 3 | hsa-miR-511-5p | hsa-miR-4446-5p | hsa-miR-4748 | | | |
| ***GMEB2*** | 0.000128 | 0.0693 | 4 | hsa-miR-3180-3p | hsa-miR-3180 | **hsa-miR-4488** | hsa-miR-766-5p | | |
| ***PCDHB2*** | 0.000205 | 0.041 | 3 | **hsa-miR-4488** | hsa-miR-3180-3p | hsa-miR-3180 | | | |
| ***RASD1*** | 0.000167 | 0.0385 | 3 | **hsa-miR-4488** | hsa-miR-3180-3p | hsa-miR-3180 | | | |
| ***STMN3*** | 0.000237 | 0.0808 | 4 | **hsa-miR-4488** | hsa-miR-3180-3p | hsa-miR-3180 | hsa-miR-766-5p | | |
| ***SIGLEC12*** | 0.000296 | 0.0462 | 3 | **hsa-miR-4488** | hsa-miR-3180-3p | hsa-miR-3180 | | | |
| ***LSP1*** | 0.000409 | 0.0513 | 3 | hsa-miR-3180-3p | hsa-miR-3180 | **hsa-miR-4488** | | | |
| ***MAPK8IP2*** | 0.000547 | 0.0564 | 3 | **hsa-miR-4488** | hsa-miR-3180-3p | hsa-miR-3180 | | | |
| ***MRPL44*** | 0.00063 | 0.104 | 4 | hsa-miR-3180-3p | hsa-miR-3180 | **hsa-miR-4488** | hsa-miR-4725-3p | | |
| ***VPS51*** | 0.000626 | 0.059 | 3 | **hsa-miR-4488** | hsa-miR-3180-3p | hsa-miR-3180 | | | |
| ***TACC3*** | 0.000805 | 0.0641 | 3 | **hsa-miR-4488** | hsa-miR-3180-3p | hsa-miR-3180 | | | |
| ***SALL4*** | 0.00101 | 0.0693 | 3 | hsa-miR-3180-3p | hsa-miR-3180 | **hsa-miR-4488** | | | |
| *HMX1* | 0.00113 | 0.0718 | 3 | hsa-miR-766-5p | hsa-miR-4448 | hsa-miR-6772-3p | | | |
| ***RUNX3*** | 0.00128 | 0.125 | 4 | hsa-miR-766-5p | **hsa-miR-4488** | hsa-miR-3180-3p | hsa-miR-3180 | | |
| ***CBARP*** | 0.00139 | 0.077 | 3 | **hsa-miR-4488** | hsa-miR-3180-3p | hsa-miR-3180 | | | |
| *LY6K* | 0.002 | 0.0872 | 3 | hsa-miR-6772-3p | hsa-miR-4448 | hsa-miR-6837-3p | | | |
| *SEMA3G* | 0.00196 | 0.0346 | 2 | hsa-miR-6772-3p | hsa-miR-4448 | | | | |
| *PITX3* | 0.00244 | 0.0385 | 2 | hsa-miR-3180-3p | hsa-miR-3180 | | | | |
| *ASCL2* | 0.00256 | 0.0949 | 3 | hsa-miR-3180-3p | hsa-miR-3180 | hsa-miR-766-5p | | | |
| *CASP16P* | 0.00317 | 0.16 | 4 | hsa-miR-365b-5p | hsa-miR-3180-3p | hsa-miR-3180 | hsa-miR-181d-3p | | |
| ***KDELC2*** | 0.00321 | 0.103 | 3 | hsa-miR-3180-3p | hsa-miR-3180 | **hsa-miR-4488** | | | |
| ***MSI1*** | 0.00321 | 0.103 | 3 | **hsa-miR-4488** | hsa-miR-3180-3p | hsa-miR-3180 | | | |
| ***SBF1*** | 0.00331 | 0.162 | 4 | hsa-miR-3180-3p | hsa-miR-3180 | **hsa-miR-4488** | hsa-miR-4725-3p | | |
| *DGKD* | 0.00417 | 0.05 | 2 | hsa-miR-766-5p | hsa-miR-6837-3p | | | | |
| *KCNK3* | 0.00479 | 0.118 | 3 | hsa-miR-3180-3p | hsa-miR-3180 | hsa-miR-365b-5p | | | |

| | | | | | | | | | |
|---|---|---|---|---|---|---|---|---|---|
| *MARVELD1* | 0.00509 | 0.121 | 3 | hsa-miR-3180-3p | hsa-miR-3180 | hsa-miR-4725-3p | | | |
| ***MIDN*** | 0.00462 | 0.28 | 6 | hsa-miR-4725-3p | hsa-miR-4748 | **hsa-miR-4488** | hsa-miR-3180-3p | hsa-miR-3180 | hsa-miR-766-5p |
| *OTUB1* | 0.0045 | 0.115 | 3 | hsa-miR-3180-3p | hsa-miR-3180 | hsa-miR-4725-3p | | | |
| ***RXRB*** | 0.00509 | 0.121 | 3 | **hsa-miR-4488** | hsa-miR-3180-3p | hsa-miR-3180 | | | |
| *SLC5A12* | 0.0045 | 0.115 | 3 | hsa-miR-4446-5p | hsa-miR-6772-3p | hsa-miR-4448 | | | |
| *SRRM4* | 0.0045 | 0.115 | 3 | hsa-miR-511-5p | hsa-miR-6772-3p | hsa-miR-4448 | | | |
| *TBC1D14* | 0.00484 | 0.0539 | 2 | hsa-miR-4448 | hsa-miR-6772-3p | | | | |
| *VPS18* | 0.00422 | 0.113 | 3 | hsa-miR-3180-3p | hsa-miR-3180 | hsa-miR-508-3p | | | |
| *XPO6* | 0.0037 | 0.108 | 3 | hsa-miR-3180-3p | hsa-miR-3180 | hsa-miR-766-5p | | | |
| *ZCCHC3* | 0.00426 | 0.173 | 4 | hsa-miR-4725-3p | hsa-miR-181d-3p | hsa-miR-3180-3p | hsa-miR-3180 | | |
| *CHD5* | 0.00556 | 0.0577 | 2 | hsa-miR-4446-5p | hsa-miR-4748 | | | | |
| *ZNF330* | 0.00556 | 0.0577 | 2 | hsa-miR-6772-3p | hsa-miR-4448 | | | | |
| ***GNAI2*** | 0.00573 | 0.126 | 3 | hsa-miR-3180-3p | hsa-miR-3180 | **hsa-miR-4488** | | | |
| *MELTF* | 0.00633 | 0.0616 | 2 | hsa-miR-3180-3p | hsa-miR-3180 | | | | |
| *AMH* | 0.008 | 0.0693 | 2 | hsa-miR-3180-3p | hsa-miR-3180 | | | | |
| *ENG* | 0.00714 | 0.0654 | 2 | hsa-miR-3180-3p | hsa-miR-3180 | | | | |
| ***H2AFX*** | 0.00677 | 0.133 | 3 | **hsa-miR-4488** | hsa-miR-3180-3p | hsa-miR-3180 | | | |
| *HTR1F* | 0.008 | 0.0693 | 2 | hsa-miR-6772-3p | hsa-miR-4448 | | | | |
| *ISL2* | 0.008 | 0.0693 | 2 | hsa-miR-4725-3p | hsa-miR-4632-3p | | | | |
| *MARCH1* | 0.00714 | 0.0654 | 2 | hsa-miR-4446-5p | hsa-miR-4748 | | | | |
| ***NEUROD2*** | 0.00753 | 0.139 | 3 | **hsa-miR-4488** | hsa-miR-3180-3p | hsa-miR-3180 | | | |
| *ST7L* | 0.008 | 0.0693 | 2 | hsa-miR-3180-3p | hsa-miR-3180 | | | | |
| *UQCC3* | 0.008 | 0.0693 | 2 | hsa-miR-3180-3p | hsa-miR-3180 | | | | |
| ***THSD4*** | 0.00833 | 0.144 | 3 | **hsa-miR-4488** | hsa-miR-3180-3p | hsa-miR-3180 | | | |
| *SLITRK5* | 0.0089 | 0.0731 | 2 | hsa-miR-3180-3p | hsa-miR-3180 | | | | |
| *TULP1* | 0.0089 | 0.0731 | 2 | hsa-miR-3180-3p | hsa-miR-3180 | | | | |
| ***SHISA2*** | 0.00918 | 0.149 | 3 | **hsa-miR-4488** | hsa-miR-3180-3p | hsa-miR-3180 | | | |
| *SLAMF1* | 0.00984 | 0.077 | 2 | hsa-miR-6837-3p | hsa-miR-4448 | | | | |
| *TMEM117* | 0.00984 | 0.077 | 2 | hsa-miR-365b-5p | hsa-miR-2116-5p | | | | |
| *C3orf52* | 0.0108 | 0.0808 | 2 | hsa-miR-4446-5p | hsa-miR-4748 | | | | |

| | | | | | | | | | |
|---|---|---|---|---|---|---|---|---|---|
| *HMGCLL1* | 0.0108 | 0.0808 | 2 | hsa-miR-6772-3p | hsa-miR-4448 | | | | |
| ***RPL28*** | 0.011 | 0.159 | 3 | hsa-miR-3180-3p | hsa-miR-3180 | **hsa-miR-4488** | | | |
| *BICDL1* | 0.012 | 0.164 | 3 | hsa-miR-3180-3p | hsa-miR-3180 | hsa-miR-766-5p | | | |
| *CCDC83* | 0.0119 | 0.0846 | 2 | hsa-miR-511-5p | hsa-miR-4773 | | | | |
| *DECR1* | 0.0119 | 0.0846 | 2 | hsa-miR-511-5p | hsa-miR-4773 | | | | |
| *NUAK2* | 0.0119 | 0.0846 | 2 | hsa-miR-766-5p | hsa-miR-4748 | | | | |
| *PRICKLE4* | 0.012 | 0.164 | 3 | hsa-miR-4725-3p | hsa-miR-4446-5p | hsa-miR-508-3p | | | |
| *C3orf36* | 0.0131 | 0.169 | 3 | hsa-miR-3180-3p | hsa-miR-3180 | hsa-miR-4725-3p | | | |
| ***ENTPD5*** | 0.0131 | 0.169 | 3 | **hsa-miR-4488** | hsa-miR-3180-3p | hsa-miR-3180 | | | |
| *FAM43A* | 0.0129 | 0.0885 | 2 | hsa-miR-3180-3p | hsa-miR-3180 | | | | |
| *KLF9* | 0.0129 | 0.0885 | 2 | hsa-miR-511-5p | hsa-miR-4748 | | | | |
| ***SLC10A7*** | 0.0131 | 0.169 | 3 | **hsa-miR-4488** | hsa-miR-3180-3p | hsa-miR-3180 | | | |
| ***ATG2A*** | 0.0136 | 0.172 | 3 | **hsa-miR-4488** | hsa-miR-3180-3p | hsa-miR-3180 | | | |
| *CAPN5* | 0.014 | 0.0923 | 2 | hsa-miR-4448 | hsa-miR-6772-3p | | | | |
| *CRTC2* | 0.014 | 0.0923 | 2 | hsa-miR-4725-3p | hsa-miR-766-5p | | | | |
| *FANCM* | 0.014 | 0.0923 | 2 | hsa-miR-6772-3p | hsa-miR-4448 | | | | |
| *FAM193A* | 0.0152 | 0.0962 | 2 | hsa-miR-3180-3p | hsa-miR-3180 | | | | |
| ***GNB2*** | 0.0152 | 0.0962 | 2 | **hsa-miR-4488** | hsa-miR-766-5p | | | | |
| *REPIN1* | 0.0154 | 0.18 | 3 | hsa-miR-4725-3p | hsa-miR-3180-3p | hsa-miR-3180 | | | |
| ***ZNF385A*** | 0.0147 | 0.246 | 4 | hsa-miR-4725-3p | **hsa-miR-4488** | hsa-miR-3180 | hsa-miR-3180-3p | | |
| *CELF2* | 0.0176 | 0.104 | 2 | hsa-miR-4446-5p | hsa-miR-4748 | | | | |
| *CEP19* | 0.0176 | 0.104 | 2 | hsa-miR-4448 | hsa-miR-508-3p | | | | |
| *DPF1* | 0.0189 | 0.108 | 2 | hsa-miR-3180-3p | hsa-miR-3180 | | | | |
| *GMDS* | 0.0189 | 0.108 | 2 | hsa-miR-3180-3p | hsa-miR-3180 | | | | |
| *MSC* | 0.0189 | 0.108 | 2 | hsa-miR-3180-3p | hsa-miR-3180 | | | | |
| ***PAX2*** | 0.0189 | 0.108 | 2 | **hsa-miR-4488** | hsa-miR-766-5p | | | | |
| *POU3F3* | 0.0189 | 0.108 | 2 | hsa-miR-3180-3p | hsa-miR-3180 | | | | |
| ***RAB11B*** | 0.0185 | 0.192 | 3 | **hsa-miR-4488** | hsa-miR-3180-3p | hsa-miR-3180 | | | |
| *RASSF8* | 0.0189 | 0.108 | 2 | hsa-miR-511-5p | hsa-miR-508-3p | | | | |
| *SEC24A* | 0.0185 | 0.192 | 3 | hsa-miR-4748 | hsa-miR-2116-5p | hsa-miR-6837-3p | | | |

| | | | | | | | | | |
|---|---|---|---|---|---|---|---|---|---|
| *SET* | 0.0178 | 0.19 | 3 | hsa-miR-1185-2-3p | hsa-miR-766-5p | hsa-miR-6772-3p | | | |
| *C10orf55* | 0.0202 | 0.112 | 2 | hsa-miR-3180-3p | hsa-miR-3180 | | | | |
| *NOX5* | 0.0202 | 0.112 | 2 | hsa-miR-4748 | hsa-miR-4446-5p | | | | |
| *RREB1* | 0.0194 | 0.267 | 4 | hsa-miR-4725-3p | hsa-miR-4773 | hsa-miR-6772-3p | hsa-miR-4448 | | |
| *TMEM242* | 0.0202 | 0.112 | 2 | hsa-miR-6837-3p | hsa-miR-4748 | | | | |
| *TTC38* | 0.0202 | 0.112 | 2 | hsa-miR-4446-5p | hsa-miR-365b-5p | | | | |
| *ZBTB7B* | 0.0205 | 0.2 | 3 | hsa-miR-4632-3p | hsa-miR-3180-3p | hsa-miR-3180 | | | |
| *CAND1* | 0.0242 | 0.213 | 3 | hsa-miR-1185-2-3p | hsa-miR-511-5p | hsa-miR-2116-5p | | | |
| *CD99* | 0.0243 | 0.123 | 2 | hsa-miR-1185-2-3p | hsa-miR-365b-5p | | | | |
| *CLCN7* | 0.0229 | 0.119 | 2 | hsa-miR-3180-3p | hsa-miR-3180 | | | | |
| *CSK* | 0.0243 | 0.123 | 2 | hsa-miR-3180-3p | hsa-miR-3180 | | | | |
| *EFHD2* | 0.0242 | 0.213 | 3 | hsa-miR-4748 | hsa-miR-6772-3p | hsa-miR-766-5p | | | |
| *LEPROTL1* | 0.0243 | 0.123 | 2 | hsa-miR-3180-3p | hsa-miR-3180 | | | | |
| *MIA3* | 0.0243 | 0.123 | 2 | hsa-miR-6772-3p | hsa-miR-4448 | | | | |
| *OLR1* | 0.0227 | 0.208 | 3 | hsa-miR-4725-3p | hsa-miR-3180-3p | hsa-miR-3180 | | | |
| *PBX2* | 0.0215 | 0.115 | 2 | hsa-miR-3180-3p | hsa-miR-3180 | | | | |
| *PFKFB2* | 0.0229 | 0.119 | 2 | hsa-miR-1185-2-3p | hsa-miR-4725-3p | | | | |
| *PRKCD* | 0.0243 | 0.123 | 2 | hsa-miR-6772-3p | hsa-miR-4448 | | | | |
| *CSNK1D* | 0.0258 | 0.127 | 2 | hsa-miR-4448 | hsa-miR-6772-3p | | | | |
| *KANSL1L* | 0.0258 | 0.127 | 2 | hsa-miR-4748 | hsa-miR-1185-2-3p | | | | |
| *PLEKHO1* | 0.0258 | 0.127 | 2 | hsa-miR-6772-3p | hsa-miR-4448 | | | | |
| ***FEM1A*** | 0.0262 | 0.352 | 5 | hsa-miR-4725-3p | **hsa-miR-4488** | hsa-miR-3180-3p | hsa-miR-3180 | hsa-miR-365b-5p | |
| *AZIN1* | 0.0272 | 0.131 | 2 | hsa-miR-2116-5p | hsa-miR-4725-3p | | | | |
| *C1orf52* | 0.0272 | 0.131 | 2 | hsa-miR-1185-2-3p | hsa-miR-2116-5p | | | | |
| *IER3* | 0.0272 | 0.131 | 2 | hsa-miR-3942-5p | hsa-miR-766-5p | | | | |
| *INTU* | 0.0274 | 0.223 | 3 | hsa-miR-3942-5p | hsa-miR-4773 | hsa-miR-365b-5p | | | |
| *AMOTL2* | 0.029 | 0.228 | 3 | hsa-miR-4446-5p | hsa-miR-6772-3p | hsa-miR-4448 | | | |
| ***LYRM4*** | 0.0299 | 0.231 | 3 | **hsa-miR-4488** | hsa-miR-3180-3p | hsa-miR-3180 | | | |
| *PKNOX2* | 0.0288 | 0.135 | 2 | hsa-miR-1185-2-3p | hsa-miR-4725-3p | | | | |
| *YAF2* | 0.0288 | 0.135 | 2 | hsa-miR-2116-5p | hsa-miR-3942-5p | | | | |

| | | | | | | | | | |
|---|---|---|---|---|---|---|---|---|---|
| *RHOB* | 0.0316 | 0.236 | 3 | hsa-miR-4446-5p | hsa-miR-3180-3p | hsa-miR-3180 | | | |
| *IL21R* | 0.0319 | 0.142 | 2 | hsa-miR-4748 | hsa-miR-766-5p | | | | |
| *SELENON* | 0.0325 | 0.239 | 3 | hsa-miR-6772-3p | hsa-miR-4448 | hsa-miR-6837-3p | | | |
| *SCAMP3* | 0.0335 | 0.146 | 2 | hsa-miR-3180-3p | hsa-miR-3180 | | | | |
| *TFAP2B* | 0.0335 | 0.146 | 2 | hsa-miR-4632-3p | hsa-miR-181d-3p | | | | |
| *UNK* | 0.0344 | 0.244 | 3 | hsa-miR-3180-3p | hsa-miR-3180 | hsa-miR-4725-3p | | | |
| *POU3F1* | 0.0352 | 0.15 | 2 | hsa-miR-3180-3p | hsa-miR-3180 | | | | |
| *ZBTB22* | 0.0352 | 0.15 | 2 | hsa-miR-6772-3p | hsa-miR-4448 | | | | |
| ***GPAT4*** | 0.0372 | 0.251 | 3 | **hsa-miR-4488** | hsa-miR-3180-3p | hsa-miR-3180 | | | |
| *RAD50* | 0.0368 | 0.154 | 2 | hsa-miR-766-5p | hsa-miR-511-5p | | | | |
| *MTA1* | 0.0386 | 0.158 | 2 | hsa-miR-3180-3p | hsa-miR-3180 | | | | |
| *SGCD* | 0.0386 | 0.158 | 2 | hsa-miR-511-5p | hsa-miR-365b-5p | | | | |
| ***CERS1*** | 0.0403 | 0.162 | 2 | hsa-miR-4632-3p | **hsa-miR-4488** | | | | |
| *GBA2* | 0.0403 | 0.162 | 2 | hsa-miR-4725-3p | hsa-miR-766-5p | | | | |
| *MAX* | 0.0403 | 0.162 | 2 | hsa-miR-1185-2-3p | hsa-miR-4725-3p | | | | |
| *PCTP* | 0.0403 | 0.162 | 2 | hsa-miR-4446-5p | hsa-miR-1185-2-3p | | | | |
| *RNF144B* | 0.0403 | 0.162 | 2 | hsa-miR-3180-3p | hsa-miR-3180 | | | | |
| *RNF157* | 0.0392 | 0.257 | 3 | hsa-miR-766-5p | hsa-miR-6772-3p | hsa-miR-4448 | | | |
| *MARCH9* | 0.0421 | 0.165 | 2 | hsa-miR-4725-3p | hsa-miR-181d-3p | | | | |
| *ROBO1* | 0.0421 | 0.165 | 2 | hsa-miR-1185-2-3p | hsa-miR-508-3p | | | | |
| ***CS*** | 0.0457 | 0.173 | 2 | **hsa-miR-4488** | hsa-miR-766-5p | | | | |
| *EXOC5* | 0.0439 | 0.169 | 2 | hsa-miR-4773 | hsa-miR-4446-5p | | | | |
| *NOVA2* | 0.0439 | 0.169 | 2 | hsa-miR-766-5p | hsa-miR-4446-5p | | | | |
| *NRGN* | 0.0439 | 0.169 | 2 | hsa-miR-3180-3p | hsa-miR-3180 | | | | |
| *OIP5* | 0.0439 | 0.169 | 2 | hsa-miR-365b-5p | hsa-miR-508-3p | | | | |
| *SESN2* | 0.0443 | 0.269 | 3 | hsa-miR-4748 | hsa-miR-4725-3p | hsa-miR-766-5p | | | |
| *UTP15* | 0.0457 | 0.173 | 2 | hsa-miR-6772-3p | hsa-miR-4448 | | | | |
| *ZNF260* | 0.0439 | 0.169 | 2 | hsa-miR-4773 | hsa-miR-6837-3p | | | | |
| *ARAP2* | 0.0476 | 0.177 | 2 | hsa-miR-1185-2-3p | hsa-miR-4446-5p | | | | |
| ***MSN*** | 0.0475 | 0.277 | 3 | hsa-miR-6772-3p | hsa-miR-4448 | **hsa-miR-4488** | | | |

| | | | | | | | | | |
|---|---|---|---|---|---|---|---|---|---|
| *ZNF444* | 0.0476 | 0.177 | 2 | hsa-miR-766-5p | hsa-miR-4725-3p | | | | |
| *KSR2* | 0.0495 | 0.181 | 2 | hsa-miR-766-5p | hsa-miR-511-5p | | | | |
| *MTRNR2L10* | 0.0514 | 0.185 | 2 | hsa-miR-508-3p | hsa-miR-2116-5p | | | | |
| *RCAN1* | 0.0495 | 0.181 | 2 | hsa-miR-4446-5p | hsa-miR-2116-5p | | | | |
| *SLC9A7* | 0.0495 | 0.181 | 2 | hsa-miR-3180-3p | hsa-miR-3180 | | | | |
| *WNK1* | 0.0498 | 0.282 | 3 | hsa-miR-1185-2-3p | hsa-miR-6772-3p | hsa-miR-4448 | | | |

Analyses were performed using Mienturnet (DB: miRTarBase), and only statistically significant genes ($p < 0.05$) are presented. Gene symbols corresponding to hsa-miR-4488, which was downregulated in both the high Pb and ISS groups, are indicated in bold.

**Table S6.** Predicted target genes of differentially expressed miRNAs in the high urinary As group compared with the low As group.

| Gene symbol | p-value | Odd ratio | Number of interactions | microRNA 1 | microRNA 2 | microRNA 3 |
|---|---|---|---|---|---|---|
| ***ALDH9A1*** | 0.02 | 0.114 | 2 | hsa-miR-3614-5p | **hsa-miR-4516** | |
| ***C11orf24*** | 0.00523 | 0.0569 | 2 | **hsa-miR-133a-3p** | hsa-miR-3614-5p | |
| *CALHM5* | 0.0255 | 0.129 | 2 | hsa-miR-4676-3p | hsa-miR-3183 | |
| ***CCDC39*** | 0.00186 | 0.0339 | 2 | **hsa-miR-133a-3p** | hsa-miR-4676-3p | |
| ***CMTM4*** | 0.0122 | 0.0877 | 2 | **hsa-miR-4516** | **hsa-miR-133a-3p** | |
| *EIF5AL1* | 0.0456 | 0.177 | 2 | hsa-miR-4488 | **hsa-miR-4516** | |
| *FBRS* | 0.0346 | 0.152 | 2 | hsa-miR-3614-5p | hsa-miR-4488 | |
| *HACE1* | 0.0238 | 0.125 | 2 | hsa-miR-4676-3p | hsa-miR-3614-5p | |
| ***KCNH2*** | 0.00907 | 0.0754 | 2 | **hsa-miR-133a-3p** | **hsa-miR-4516** | |
| ***KPNA6*** | 0.0464 | 0.179 | 2 | **hsa-miR-4516** | **hsa-miR-133a-3p** | |
| ***MC2R*** | 0.013 | 0.0908 | 2 | hsa-miR-3614-5p | **hsa-miR-133a-3p** | |
| *MED4* | 0.00346 | 0.0462 | 2 | hsa-miR-1246 | hsa-miR-4676-3p | |
| ***MNT*** | 0.04 | 0.165 | 2 | hsa-miR-3614-5p | **hsa-miR-4516** | |
| ***MSN*** | 0.0407 | 0.166 | 2 | **hsa-miR-133a-3p** | hsa-miR-4488 | |
| *NACC1* | 0.0426 | 0.279 | 3 | hsa-miR-4488 | hsa-miR-3183 | hsa-miR-3614-5p |
| ***NF2*** | 0.0379 | 0.16 | 2 | hsa-miR-4488 | **hsa-miR-4516** | |
| ***NFAM1*** | 0.0061 | 0.0616 | 2 | hsa-miR-3183 | **hsa-miR-133a-3p** | |
| *PIM1* | 0.00703 | 0.0662 | 2 | **hsa-miR-4516** | hsa-miR-1246 | |
| *POLR3D* | 0.0147 | 0.097 | 2 | hsa-miR-3183 | hsa-miR-3614-5p | |
| ***POU3F1*** | 0.0058 | 0.06 | 2 | hsa-miR-3183 | **hsa-miR-4516** | |
| ***PTPN14*** | 0.0464 | 0.179 | 2 | **hsa-miR-4516** | hsa-miR-3183 | |
| *RAB11B* | 0.0206 | 0.115 | 2 | hsa-miR-4488 | hsa-miR-6821-5p | |
| ***REST*** | 0.0353 | 0.154 | 2 | **hsa-miR-4516** | hsa-miR-4676-3p | |
| *SERPINA3* | 0.00186 | 0.0339 | 2 | hsa-miR-3183 | hsa-miR-3614-5p | |
| ***SLC10A6*** | 0.0407 | 0.166 | 2 | hsa-miR-3614-5p | **hsa-miR-4516** | |
| *SLC12A5* | 0.00552 | 0.0585 | 2 | hsa-miR-4488 | hsa-miR-3614-5p | |
| *SLC16A5* | 0.00552 | 0.0585 | 2 | hsa-miR-3614-5p | hsa-miR-3183 | |
| ***SLC7A5*** | 0.041 | 0.275 | 3 | **hsa-miR-4516** | hsa-miR-4488 | hsa-miR-6821-5p |
| ***THBS2*** | 0.0238 | 0.125 | 2 | **hsa-miR-133a-3p** | hsa-miR-3183 | |
| *TMCO1* | 0.0386 | 0.162 | 2 | hsa-miR-3183 | hsa-miR-3614-5p | |
| ***TRIM71*** | 0.0379 | 0.16 | 2 | hsa-miR-3614-5p | **hsa-miR-133a-3p** | |
| *ZNF799* | 0.018 | 0.108 | 2 | hsa-miR-3614-5p | hsa-miR-4676-3p | |
| *ZNF85* | 0.0216 | 0.119 | 2 | hsa-miR-1246 | hsa-miR-3183 | |

Analyses were performed using Mienturnet (DB: miRTarBase), and only statistically significant genes ($p < 0.05$) are presented. Gene symbols corresponding to hsa-miR-4516 and hsa-miR-133a-3p, which were upregulated in both the high As and GHD groups, are indicated in bold.

**Table S7.** Predicted target genes of differentially expressed miRNAs in the high urinary Hg group compared with the low Hg group.

| Gene symbol | p-value | Odd ratio | Number of interactions | microRNA 1 | microRNA 2 | microRNA 3 | microRNA 4 | microRNA 5 |
|---|---|---|---|---|---|---|---|---|
| ***TOR4A*** | 0.000154 | 0.0762 | 4 | **hsa-miR-4516** | hsa-miR-1234-3p | hsa-miR-3180-3p | hsa-miR-3180 | |
| *VPS51* | 0.0000955 | 0.0324 | 3 | hsa-miR-3180-3p | hsa-miR-3180 | hsa-miR-6724-5p | | |
| *CASP16P* | 0.000269 | 0.0878 | 4 | hsa-miR-1234-3p | hsa-miR-3180-3p | hsa-miR-3180 | hsa-miR-6807-5p | |
| *AMH* | 0.0024 | 0.0381 | 2 | hsa-miR-3180-3p | hsa-miR-3180 | | | |
| *ATG2A* | 0.00233 | 0.0945 | 3 | hsa-miR-3180-3p | hsa-miR-3180 | hsa-miR-376b-3p | | |
| ***BCL9L*** | 0.00402 | 0.114 | 3 | hsa-miR-3180-3p | hsa-miR-3180 | **hsa-miR-4516** | | |
| *BHMT2* | 0.00233 | 0.0945 | 3 | hsa-miR-3180-3p | hsa-miR-3180 | hsa-miR-1234-3p | | |
| *CD180* | 0.00348 | 0.109 | 3 | hsa-miR-6807-5p | hsa-miR-3614-5p | hsa-miR-450a-1-3p | | |
| *CD96* | 0.0024 | 0.0381 | 2 | hsa-miR-1234-3p | hsa-miR-6807-5p | | | |
| *COL4A3BP* | 0.00476 | 0.121 | 3 | hsa-miR-6807-5p | hsa-miR-3614-5p | hsa-miR-450a-1-3p | | |
| *DPF1* | 0.0058 | 0.0593 | 2 | hsa-miR-3180-3p | hsa-miR-3180 | | | |
| *ENG* | 0.00214 | 0.036 | 2 | hsa-miR-3180-3p | hsa-miR-3180 | | | |
| *EVI5* | 0.00431 | 0.117 | 3 | hsa-miR-450a-1-3p | hsa-miR-3614-5p | hsa-miR-6807-5p | | |
| *FAM193A* | 0.00463 | 0.0529 | 2 | hsa-miR-3180-3p | hsa-miR-3180 | | | |
| *FAM43A* | 0.00393 | 0.0487 | 2 | hsa-miR-3180-3p | hsa-miR-3180 | | | |
| *FBXO6* | 0.000878 | 0.0233 | 2 | hsa-miR-6807-5p | hsa-miR-1234-3p | | | |
| *FOXK1* | 0.00264 | 0.219 | 5 | hsa-miR-6807-5p | hsa-miR-376b-3p | hsa-miR-3180-3p | hsa-miR-3180 | hsa-miR-6724-5p |
| *FXYD1* | 0.0058 | 0.0593 | 2 | hsa-miR-3180-3p | hsa-miR-3180 | | | |
| *GMDS* | 0.0058 | 0.0593 | 2 | hsa-miR-3180-3p | hsa-miR-3180 | | | |
| *LSP1* | 0.00297 | 0.0423 | 2 | hsa-miR-3180-3p | hsa-miR-3180 | | | |
| *LYRM4* | 0.00542 | 0.127 | 3 | hsa-miR-6807-5p | hsa-miR-3180-3p | hsa-miR-3180 | | |
| *MAPK8IP2* | 0.00359 | 0.0466 | 2 | hsa-miR-3180-3p | hsa-miR-3180 | | | |
| *MELTF* | 0.00189 | 0.0339 | 2 | hsa-miR-3180-3p | hsa-miR-3180 | | | |
| *METTL14* | 0.00431 | 0.117 | 3 | hsa-miR-450a-1-3p | hsa-miR-6807-5p | hsa-miR-1234-3p | | |
| *MSC* | 0.0058 | 0.0593 | 2 | hsa-miR-3180-3p | hsa-miR-3180 | | | |
| *PCDHB2* | 0.00189 | 0.0339 | 2 | hsa-miR-3180-3p | hsa-miR-3180 | | | |
| *PDS5A* | 0.0054 | 0.0571 | 2 | hsa-miR-2115-3p | hsa-miR-376b-3p | | | |

| | | | | | | | | |
|---|---|---|---|---|---|---|---|---|
| *PITX3* | 0.00072 | 0.0212 | 2 | hsa-miR-3180-3p | hsa-miR-3180 | | | |
| ***POU3F1*** | 0.000475 | 0.055 | 3 | hsa-miR-3180-3p | hsa-miR-3180 | **hsa-miR-4516** | | |
| *POU3F3* | 0.0058 | 0.0593 | 2 | hsa-miR-3180-3p | hsa-miR-3180 | | | |
| *RAB11B* | 0.00322 | 0.106 | 3 | hsa-miR-3180-3p | hsa-miR-3180 | hsa-miR-6807-5p | | |
| *RASD1* | 0.00166 | 0.0317 | 2 | hsa-miR-3180-3p | hsa-miR-3180 | | | |
| *RBM4B* | 0.00446 | 0.119 | 3 | hsa-miR-6807-5p | hsa-miR-1234-3p | hsa-miR-3614-5p | | |
| *RHOB* | 0.00576 | 0.13 | 3 | hsa-miR-2115-3p | hsa-miR-3180-3p | hsa-miR-3180 | | |
| *RNF144B* | 0.000592 | 0.0593 | 3 | hsa-miR-6807-5p | hsa-miR-3180-3p | hsa-miR-3180 | | |
| *RPL24* | 0.00214 | 0.0917 | 3 | hsa-miR-1234-3p | hsa-miR-450a-1-3p | hsa-miR-3614-5p | | |
| *SALL4* | 0.0054 | 0.0571 | 2 | hsa-miR-3180-3p | hsa-miR-3180 | | | |
| ***SFTPB*** | 0.0054 | 0.0571 | 2 | hsa-miR-6807-5p | **hsa-miR-133a-3p** | | | |
| *SHCBP1* | 0.0054 | 0.0571 | 2 | hsa-miR-3614-5p | hsa-miR-2115-3p | | | |
| *SHISA2* | 0.00153 | 0.0818 | 3 | hsa-miR-3180-3p | hsa-miR-3180 | hsa-miR-450a-1-3p | | |
| *SIGLEC12* | 0.0024 | 0.0381 | 2 | hsa-miR-3180-3p | hsa-miR-3180 | | | |
| *SLITRK5* | 0.00268 | 0.0402 | 2 | hsa-miR-3180-3p | hsa-miR-3180 | | | |
| *SMURF2* | 0.0058 | 0.0593 | 2 | hsa-miR-6807-5p | hsa-miR-1234-3p | | | |
| *ST7L* | 0.0024 | 0.0381 | 2 | hsa-miR-3180-3p | hsa-miR-3180 | | | |
| ***STAT3*** | 0.00388 | 0.113 | 3 | **hsa-miR-4516** | hsa-miR-1234-3p | hsa-miR-6807-5p | | |
| *TACC3* | 0.00463 | 0.0529 | 2 | hsa-miR-3180-3p | hsa-miR-3180 | | | |
| *TULP1* | 0.00268 | 0.0402 | 2 | hsa-miR-3180-3p | hsa-miR-3180 | | | |
| *UQCC3* | 0.0024 | 0.0381 | 2 | hsa-miR-3180-3p | hsa-miR-3180 | | | |
| ***VKORC1*** | 0.00124 | 0.0275 | 2 | **hsa-miR-133a-3p** | hsa-miR-6807-5p | | | |
| *VPS18* | 0.00068 | 0.0621 | 3 | hsa-miR-3180-3p | hsa-miR-3180 | hsa-miR-6807-5p | | |
| *WSCD1* | 0.000878 | 0.0233 | 2 | hsa-miR-3180-3p | hsa-miR-3180 | | | |
| *ZC3H8* | 0.00427 | 0.0508 | 2 | hsa-miR-3614-5p | hsa-miR-6807-5p | | | |
| *ZCCHC3* | 0.00542 | 0.127 | 3 | hsa-miR-3180-3p | hsa-miR-3180 | hsa-miR-6724-5p | | |
| *ZFAND4* | 0.00576 | 0.13 | 3 | hsa-miR-3614-5p | hsa-miR-450a-1-3p | hsa-miR-6807-5p | | |
| ***ZFP91*** | 0.00348 | 0.109 | 3 | hsa-miR-6807-5p | **hsa-miR-4516** | hsa-miR-450a-1-3p | | |
| *ZNF699* | 0.00287 | 0.102 | 3 | hsa-miR-376b-3p | hsa-miR-1234-3p | hsa-miR-3614-5p | | |
| *C10orf55* | 0.00621 | 0.0614 | 2 | hsa-miR-3180-3p | hsa-miR-3180 | | | |

| | | | | | | | | |
|---|---|---|---|---|---|---|---|---|
| ***FOSL2*** | 0.00612 | 0.133 | 3 | hsa-miR-6807-5p | **hsa-miR-133a-3p** | hsa-miR-450a-1-3p | | |
| *CBARP* | 0.00664 | 0.0635 | 2 | hsa-miR-3180-3p | hsa-miR-3180 | | | |
| *PBX2* | 0.00664 | 0.0635 | 2 | hsa-miR-3180-3p | hsa-miR-3180 | | | |
| *CLCN7* | 0.00708 | 0.0656 | 2 | hsa-miR-3180-3p | hsa-miR-3180 | | | |
| *EBNA1BP2* | 0.00688 | 0.138 | 3 | hsa-miR-6807-5p | hsa-miR-3614-5p | hsa-miR-450a-1-3p | | |
| ***FSCN1*** | 0.00708 | 0.14 | 3 | **hsa-miR-133a-3p** | hsa-miR-3180-3p | hsa-miR-3180 | | |
| *FTO* | 0.00708 | 0.0656 | 2 | hsa-miR-450a-1-3p | hsa-miR-6807-5p | | | |
| *CSK* | 0.00754 | 0.0677 | 2 | hsa-miR-3180-3p | hsa-miR-3180 | | | |
| *LEPROTL1* | 0.00754 | 0.0677 | 2 | hsa-miR-3180-3p | hsa-miR-3180 | | | |
| *CHCHD4* | 0.00849 | 0.072 | 2 | hsa-miR-6807-5p | hsa-miR-6724-5p | | | |
| ***PALD1*** | 0.00801 | 0.0698 | 2 | hsa-miR-1234-3p | **hsa-miR-4516** | | | |
| ***TRIM71*** | 0.00812 | 0.147 | 3 | hsa-miR-2115-3p | hsa-miR-3614-5p | **hsa-miR-133a-3p** | | |
| ***MNT*** | 0.00879 | 0.151 | 3 | hsa-miR-3614-5p | hsa-miR-6807-5p | **hsa-miR-4516** | | |
| *CA6* | 0.00898 | 0.0741 | 2 | hsa-miR-3614-5p | hsa-miR-6807-5p | | | |
| ***SLC10A6*** | 0.00902 | 0.152 | 3 | hsa-miR-450a-1-3p | hsa-miR-3614-5p | **hsa-miR-4516** | | |
| *GMEB2* | 0.00949 | 0.0762 | 2 | hsa-miR-3180-3p | hsa-miR-3180 | | | |
| *LRRC27* | 0.00949 | 0.0762 | 2 | hsa-miR-6807-5p | hsa-miR-1234-3p | | | |
| *ZKSCAN3* | 0.00949 | 0.0762 | 2 | hsa-miR-6724-5p | hsa-miR-450a-1-3p | | | |
| *ACOX1* | 0.0102 | 0.159 | 3 | hsa-miR-450a-1-3p | hsa-miR-6807-5p | hsa-miR-1234-3p | | |
| *ASCL2* | 0.01 | 0.0783 | 2 | hsa-miR-3180-3p | hsa-miR-3180 | | | |
| ***C11orf24*** | 0.01 | 0.0783 | 2 | **hsa-miR-133a-3p** | hsa-miR-3614-5p | | | |
| ***LRIG2*** | 0.0102 | 0.159 | 3 | **hsa-miR-4516** | hsa-miR-6807-5p | hsa-miR-450a-1-3p | | |
| *PARP2* | 0.0105 | 0.161 | 3 | hsa-miR-3614-5p | hsa-miR-450a-1-3p | hsa-miR-6807-5p | | |
| *PRIM1* | 0.00973 | 0.157 | 3 | hsa-miR-6807-5p | hsa-miR-3614-5p | hsa-miR-1234-3p | | |
| *RHD* | 0.01 | 0.0783 | 2 | hsa-miR-3614-5p | hsa-miR-6807-5p | | | |
| *SCAMP3* | 0.0105 | 0.0804 | 2 | hsa-miR-3180-3p | hsa-miR-3180 | | | |
| *ZNF584* | 0.0105 | 0.161 | 3 | hsa-miR-450a-1-3p | hsa-miR-3614-5p | hsa-miR-1234-3p | | |
| ***KPNA6*** | 0.011 | 0.164 | 3 | hsa-miR-6807-5p | **hsa-miR-4516** | **hsa-miR-133a-3p** | | |
| ***PSMG1*** | 0.0111 | 0.0825 | 2 | hsa-miR-6807-5p | **hsa-miR-133a-3p** | | | |
| *KDELC2* | 0.0116 | 0.0846 | 2 | hsa-miR-3180-3p | hsa-miR-3180 | | | |

| | | | | | | | | |
|---|---|---|---|---|---|---|---|---|
| *MLH1* | 0.0116 | 0.0846 | 2 | hsa-miR-1234-3p | hsa-miR-450a-1-3p | | | |
| *MSI1* | 0.0116 | 0.0846 | 2 | hsa-miR-3180-3p | hsa-miR-3180 | | | |
| *MTA1* | 0.0122 | 0.0868 | 2 | hsa-miR-3180-3p | hsa-miR-3180 | | | |
| *STMN3* | 0.0128 | 0.0889 | 2 | hsa-miR-3180-3p | hsa-miR-3180 | | | |
| *TRUB2* | 0.0121 | 0.169 | 3 | hsa-miR-450a-1-3p | hsa-miR-6807-5p | hsa-miR-3614-5p | | |
| *TTLL1* | 0.0128 | 0.0889 | 2 | hsa-miR-6807-5p | hsa-miR-450a-1-3p | | | |
| *XPO6* | 0.0128 | 0.0889 | 2 | hsa-miR-3180-3p | hsa-miR-3180 | | | |
| *MRNIP* | 0.0132 | 0.175 | 3 | hsa-miR-6807-5p | hsa-miR-450a-1-3p | hsa-miR-3614-5p | | |
| *NRGN* | 0.014 | 0.0931 | 2 | hsa-miR-3180-3p | hsa-miR-3180 | | | |
| ***SMIM14*** | 0.014 | 0.0931 | 2 | **hsa-miR-133a-3p** | hsa-miR-6807-5p | | | |
| *OTUB1* | 0.0146 | 0.0952 | 2 | hsa-miR-3180-3p | hsa-miR-3180 | | | |
| *SPEM1* | 0.0146 | 0.0952 | 2 | hsa-miR-1234-3p | hsa-miR-6807-5p | | | |
| *GNG4* | 0.0152 | 0.0973 | 2 | hsa-miR-450a-1-3p | hsa-miR-6807-5p | | | |
| *KCNK3* | 0.0152 | 0.0973 | 2 | hsa-miR-3180-3p | hsa-miR-3180 | | | |
| *MARVELD1* | 0.0159 | 0.0995 | 2 | hsa-miR-3180-3p | hsa-miR-3180 | | | |
| *RXRB* | 0.0159 | 0.0995 | 2 | hsa-miR-3180-3p | hsa-miR-3180 | | | |
| *SLC9A7* | 0.0159 | 0.0995 | 2 | hsa-miR-3180-3p | hsa-miR-3180 | | | |
| *TMEM216* | 0.0159 | 0.0995 | 2 | hsa-miR-3614-5p | hsa-miR-6807-5p | | | |
| *ZNF280C* | 0.0159 | 0.0995 | 2 | hsa-miR-450a-1-3p | hsa-miR-6807-5p | | | |
| ***CCDC80*** | 0.0166 | 0.19 | 3 | **hsa-miR-4516** | hsa-miR-450a-1-3p | hsa-miR-6807-5p | | |
| *GNAI2* | 0.0172 | 0.104 | 2 | hsa-miR-3180-3p | hsa-miR-3180 | | | |
| *HAUS3* | 0.0173 | 0.193 | 3 | hsa-miR-450a-1-3p | hsa-miR-3614-5p | hsa-miR-6807-5p | | |
| ***KCNH2*** | 0.0172 | 0.104 | 2 | **hsa-miR-133a-3p** | **hsa-miR-4516** | | | |
| *PRPS1* | 0.0165 | 0.102 | 2 | hsa-miR-6807-5p | hsa-miR-3614-5p | | | |
| *ZNF791* | 0.0172 | 0.104 | 2 | hsa-miR-6807-5p | hsa-miR-376b-3p | | | |
| *GNB5* | 0.0179 | 0.106 | 2 | hsa-miR-6807-5p | hsa-miR-1234-3p | | | |
| ***NAB2*** | 0.0179 | 0.106 | 2 | **hsa-miR-4516** | hsa-miR-6807-5p | | | |
| *PNMA2* | 0.0179 | 0.106 | 2 | hsa-miR-450a-1-3p | hsa-miR-3614-5p | | | |
| *RAB42* | 0.0179 | 0.106 | 2 | hsa-miR-6807-5p | hsa-miR-2115-3p | | | |
| *CYP27C1* | 0.0185 | 0.108 | 2 | hsa-miR-376b-3p | hsa-miR-6807-5p | | | |

| | | | | | | | | |
|---|---|---|---|---|---|---|---|---|
| *GNE* | 0.0214 | 0.116 | 2 | hsa-miR-1234-3p | hsa-miR-3614-5p | | | |
| *GP5* | 0.0207 | 0.114 | 2 | hsa-miR-450a-1-3p | hsa-miR-3614-5p | | | |
| *H2AFX* | 0.0192 | 0.11 | 2 | hsa-miR-3180-3p | hsa-miR-3180 | | | |
| *KCTD21* | 0.0207 | 0.114 | 2 | hsa-miR-6807-5p | hsa-miR-1234-3p | | | |
| *MAPK8IP3* | 0.02 | 0.112 | 2 | hsa-miR-3180-3p | hsa-miR-3180 | | | |
| *MRPL44* | 0.0207 | 0.114 | 2 | hsa-miR-3180-3p | hsa-miR-3180 | | | |
| ***MYPN*** | 0.0209 | 0.207 | 3 | **hsa-miR-133a-3p** | hsa-miR-1234-3p | hsa-miR-6807-5p | | |
| *NANOS1* | 0.0214 | 0.116 | 2 | hsa-miR-3180 | hsa-miR-3180-3p | | | |
| *NEUROD2* | 0.0207 | 0.114 | 2 | hsa-miR-3180-3p | hsa-miR-3180 | | | |
| *PLCXD1* | 0.02 | 0.112 | 2 | hsa-miR-6807-5p | hsa-miR-3614-5p | | | |
| *SLC25A34* | 0.0214 | 0.116 | 2 | hsa-miR-450a-1-3p | hsa-miR-3614-5p | | | |
| *SLC39A11* | 0.0207 | 0.114 | 2 | hsa-miR-3180-3p | hsa-miR-3180 | | | |
| *SLC5A5* | 0.0185 | 0.108 | 2 | hsa-miR-3614-5p | hsa-miR-6807-5p | | | |
| ***SLC7A5*** | 0.0203 | 0.284 | 4 | **hsa-miR-4516** | hsa-miR-3180-3p | hsa-miR-3180 | hsa-miR-6724-5p | |
| ***SNRPD1*** | 0.0191 | 0.2 | 3 | **hsa-miR-4516** | hsa-miR-450a-1-3p | hsa-miR-6807-5p | | |
| *SUMF2* | 0.0192 | 0.11 | 2 | hsa-miR-6724-5p | hsa-miR-450a-1-3p | | | |
| *ZNF35* | 0.0214 | 0.116 | 2 | hsa-miR-3614-5p | hsa-miR-6807-5p | | | |
| *SESTD1* | 0.0221 | 0.119 | 2 | hsa-miR-3180-3p | hsa-miR-3180 | | | |
| *THSD4* | 0.0221 | 0.119 | 2 | hsa-miR-3180-3p | hsa-miR-3180 | | | |
| *TIGAR* | 0.0221 | 0.119 | 2 | hsa-miR-3614-5p | hsa-miR-6807-5p | | | |
| *MSANTD4* | 0.0225 | 0.213 | 3 | hsa-miR-2115-3p | hsa-miR-6807-5p | hsa-miR-450a-1-3p | | |
| *ARMT1* | 0.0229 | 0.121 | 2 | hsa-miR-3614-5p | hsa-miR-1234-3p | | | |
| ***CMTM4*** | 0.0229 | 0.121 | 2 | **hsa-miR-4516** | **hsa-miR-133a-3p** | | | |
| *ESCO2* | 0.0244 | 0.125 | 2 | hsa-miR-450a-1-3p | hsa-miR-6807-5p | | | |
| ***FUS*** | 0.0244 | 0.125 | 2 | **hsa-miR-4516** | hsa-miR-6807-5p | | | |
| ***MC2R*** | 0.0244 | 0.125 | 2 | hsa-miR-3614-5p | **hsa-miR-133a-3p** | | | |
| *TMEM251* | 0.0244 | 0.125 | 2 | hsa-miR-6807-5p | hsa-miR-450a-1-3p | | | |
| *WIZ* | 0.0244 | 0.125 | 2 | hsa-miR-450a-1-3p | hsa-miR-3614-5p | | | |
| *NUDT19* | 0.0252 | 0.127 | 2 | hsa-miR-450a-1-3p | hsa-miR-6724-5p | | | |
| *PPP1R16B* | 0.0252 | 0.127 | 2 | hsa-miR-6807-5p | hsa-miR-450a-1-3p | | | |

| | | | | | | | | |
|---|---|---|---|---|---|---|---|---|
| *TMPRSS12* | 0.0252 | 0.127 | 2 | hsa-miR-450a-1-3p | hsa-miR-3614-5p | | | |
| *ZNRF3* | 0.0252 | 0.127 | 2 | hsa-miR-376b-3p | hsa-miR-3614-5p | | | |
| *BICDL1* | 0.0285 | 0.135 | 2 | hsa-miR-3180-3p | hsa-miR-3180 | | | |
| *CACNG8* | 0.0284 | 0.233 | 3 | hsa-miR-1234-3p | hsa-miR-3614-5p | hsa-miR-6807-5p | | |
| *CBX8* | 0.0293 | 0.138 | 2 | hsa-miR-3180-3p | hsa-miR-3180 | | | |
| *CD3D* | 0.0285 | 0.135 | 2 | hsa-miR-450a-1-3p | hsa-miR-6724-5p | | | |
| *EHD2* | 0.0268 | 0.131 | 2 | hsa-miR-450a-1-3p | hsa-miR-2115-3p | | | |
| *ESR2* | 0.026 | 0.129 | 2 | hsa-miR-6807-5p | hsa-miR-1234-3p | | | |
| *PCDHA6* | 0.0285 | 0.135 | 2 | hsa-miR-450a-1-3p | hsa-miR-3614-5p | | | |
| *RNF24* | 0.0268 | 0.131 | 2 | hsa-miR-450a-1-3p | hsa-miR-6807-5p | | | |
| *RPL28* | 0.0268 | 0.131 | 2 | hsa-miR-3180-3p | hsa-miR-3180 | | | |
| *RUNX3* | 0.0293 | 0.138 | 2 | hsa-miR-3180-3p | hsa-miR-3180 | | | |
| *SYNGR1* | 0.0285 | 0.135 | 2 | hsa-miR-3180-3p | hsa-miR-3180 | | | |
| *TBRG1* | 0.0293 | 0.138 | 2 | hsa-miR-3614-5p | hsa-miR-6807-5p | | | |
| ***TPM3*** | 0.0293 | 0.236 | 3 | **hsa-miR-133a-3p** | hsa-miR-3180-3p | hsa-miR-3180 | | |
| *WDR73* | 0.0285 | 0.135 | 2 | hsa-miR-6807-5p | hsa-miR-3614-5p | | | |
| *WHAMM* | 0.0285 | 0.135 | 2 | hsa-miR-450a-1-3p | hsa-miR-3614-5p | | | |
| *ZNF619* | 0.0293 | 0.138 | 2 | hsa-miR-3614-5p | hsa-miR-6807-5p | | | |
| *C3orf36* | 0.0301 | 0.14 | 2 | hsa-miR-3180-3p | hsa-miR-3180 | | | |
| *ENTPD5* | 0.0301 | 0.14 | 2 | hsa-miR-3180-3p | hsa-miR-3180 | | | |
| ***PIGR*** | 0.0301 | 0.14 | 2 | hsa-miR-450a-1-3p | **hsa-miR-133a-3p** | | | |
| *SLC10A7* | 0.0301 | 0.14 | 2 | hsa-miR-3180-3p | hsa-miR-3180 | | | |
| *ZNF500* | 0.0301 | 0.14 | 2 | hsa-miR-6807-5p | hsa-miR-3614-5p | | | |
| *APOBEC3F* | 0.031 | 0.142 | 2 | hsa-miR-3614-5p | hsa-miR-450a-1-3p | | | |
| *AQR* | 0.031 | 0.142 | 2 | hsa-miR-450a-1-3p | hsa-miR-6807-5p | | | |
| *AHR* | 0.0345 | 0.15 | 2 | hsa-miR-6807-5p | hsa-miR-450a-1-3p | | | |
| ***ALDH9A1*** | 0.0373 | 0.157 | 2 | hsa-miR-3614-5p | **hsa-miR-4516** | | | |
| *ASB16* | 0.0356 | 0.254 | 3 | hsa-miR-1234-3p | hsa-miR-3180-3p | hsa-miR-3180 | | |
| *CCL22* | 0.0345 | 0.15 | 2 | hsa-miR-450a-1-3p | hsa-miR-6807-5p | | | |
| *CENPN* | 0.0382 | 0.159 | 2 | hsa-miR-2115-3p | hsa-miR-6807-5p | | | |

| | | | | | | | | | |
|---|---|---|---|---|---|---|---|---|---|
| *FAM118A* | 0.0373 | 0.157 | 2 | hsa-miR-6807-5p | hsa-miR-3614-5p | | | | |
| *ISPD* | 0.0382 | 0.159 | 2 | hsa-miR-3614-5p | hsa-miR-450a-1-3p | | | | |
| *LEPROT* | 0.0354 | 0.152 | 2 | hsa-miR-376b-3p | hsa-miR-6807-5p | | | | |
| *LY6G5B* | 0.0373 | 0.157 | 2 | hsa-miR-450a-1-3p | hsa-miR-1234-3p | | | | |
| ***MINOS1*** | 0.0382 | 0.159 | 2 | hsa-miR-450a-1-3p | **hsa-miR-4516** | | | | |
| *NDUFA7* | 0.0336 | 0.148 | 2 | hsa-miR-6807-5p | hsa-miR-450a-1-3p | | | | |
| *NUP205* | 0.0363 | 0.154 | 2 | hsa-miR-6807-5p | hsa-miR-3614-5p | | | | |
| *PAQR5* | 0.0319 | 0.144 | 2 | hsa-miR-450a-1-3p | hsa-miR-3614-5p | | | | |
| *PLCE1* | 0.0345 | 0.15 | 2 | hsa-miR-6807-5p | hsa-miR-3614-5p | | | | |
| *PTK6* | 0.0354 | 0.152 | 2 | hsa-miR-3614-5p | hsa-miR-6807-5p | | | | |
| *RAB4A* | 0.0336 | 0.148 | 2 | hsa-miR-6807-5p | hsa-miR-450a-1-3p | | | | |
| *REPIN1* | 0.0336 | 0.148 | 2 | hsa-miR-3180-3p | hsa-miR-3180 | | | | |
| ***RFT1*** | 0.0354 | 0.152 | 2 | **hsa-miR-133a-3p** | hsa-miR-6807-5p | | | | |
| *RSBN1L* | 0.0382 | 0.159 | 2 | hsa-miR-3180-3p | hsa-miR-3180 | | | | |
| *SBK1* | 0.0363 | 0.154 | 2 | hsa-miR-3180-3p | hsa-miR-3180 | | | | |
| *SLC1A5* | 0.0326 | 0.245 | 3 | hsa-miR-6807-5p | hsa-miR-1234-3p | hsa-miR-450a-1-3p | | | |
| *SMTNL2* | 0.0319 | 0.144 | 2 | hsa-miR-450a-1-3p | hsa-miR-3614-5p | | | | |
| ***TRAF6*** | 0.0345 | 0.15 | 2 | **hsa-miR-4516** | hsa-miR-6807-5p | | | | |
| *TTC9C* | 0.0327 | 0.146 | 2 | hsa-miR-450a-1-3p | hsa-miR-6807-5p | | | | |
| *TXK* | 0.0363 | 0.154 | 2 | hsa-miR-6807-5p | hsa-miR-1234-3p | | | | |
| *ULK2* | 0.0345 | 0.15 | 2 | hsa-miR-6807-5p | hsa-miR-3614-5p | | | | |
| *ZNF516* | 0.0382 | 0.159 | 2 | hsa-miR-3180-3p | hsa-miR-3180 | | | | |
| *ZNF799* | 0.0336 | 0.148 | 2 | hsa-miR-6807-5p | hsa-miR-3614-5p | | | | |
| *ZNF844* | 0.0382 | 0.159 | 2 | hsa-miR-3614-5p | hsa-miR-376b-3p | | | | |
| *BAMBI* | 0.0391 | 0.161 | 2 | hsa-miR-450a-1-3p | hsa-miR-3614-5p | | | | |
| ***CDKN1A*** | 0.0411 | 0.35 | 4 | **hsa-miR-4516** | **hsa-miR-133a-3p** | hsa-miR-3180-3p | hsa-miR-3180 | | |
| *COX18* | 0.041 | 0.165 | 2 | hsa-miR-450a-1-3p | hsa-miR-3614-5p | | | | |
| *COX19* | 0.0401 | 0.163 | 2 | hsa-miR-6807-5p | hsa-miR-1234-3p | | | | |
| *ISY1* | 0.0391 | 0.161 | 2 | hsa-miR-6807-5p | hsa-miR-450a-1-3p | | | | |
| *MRI1* | 0.0401 | 0.163 | 2 | hsa-miR-6807-5p | hsa-miR-1234-3p | | | | |

| | | | | | | | | |
|---|---|---|---|---|---|---|---|---|
| *PHACTR4* | 0.0391 | 0.161 | 2 | hsa-miR-3180-3p | hsa-miR-3180 | | | |
| *QPCTL* | 0.041 | 0.165 | 2 | hsa-miR-6807-5p | hsa-miR-450a-1-3p | | | |
| *TACO1* | 0.0401 | 0.163 | 2 | hsa-miR-450a-1-3p | hsa-miR-6807-5p | | | |
| *ZBTB7B* | 0.041 | 0.165 | 2 | hsa-miR-3180-3p | hsa-miR-3180 | | | |
| ***ANGPT4*** | 0.042 | 0.167 | 2 | hsa-miR-450a-1-3p | **hsa-miR-133a-3p** | | | |
| ***DNAJC10*** | 0.0449 | 0.278 | 3 | **hsa-miR-4516** | hsa-miR-6807-5p | hsa-miR-450a-1-3p | | |
| *FBXW2* | 0.044 | 0.171 | 2 | hsa-miR-450a-1-3p | hsa-miR-6807-5p | | | |
| *OLR1* | 0.044 | 0.171 | 2 | hsa-miR-3180-3p | hsa-miR-3180 | | | |
| *SLC35F5* | 0.044 | 0.171 | 2 | hsa-miR-6807-5p | hsa-miR-1234-3p | | | |
| *SP2* | 0.043 | 0.169 | 2 | hsa-miR-6724-5p | hsa-miR-1234-3p | | | |
| *TCF23* | 0.043 | 0.169 | 2 | hsa-miR-6807-5p | hsa-miR-450a-1-3p | | | |
| *ZNF682* | 0.044 | 0.171 | 2 | hsa-miR-6807-5p | hsa-miR-1234-3p | | | |
| *LINC00346* | 0.046 | 0.176 | 2 | hsa-miR-450a-1-3p | hsa-miR-3614-5p | | | |
| ***LRRC58*** | 0.0461 | 0.281 | 3 | hsa-miR-376b-3p | **hsa-miR-4516** | hsa-miR-1234-3p | | |
| ***ABHD18*** | 0.048 | 0.18 | 2 | **hsa-miR-133a-3p** | hsa-miR-1234-3p | | | |
| ***AGAP9*** | 0.048 | 0.18 | 2 | hsa-miR-450a-1-3p | **hsa-miR-4516** | | | |
| ***AHCYL2*** | 0.049 | 0.182 | 2 | **hsa-miR-4516** | hsa-miR-6807-5p | | | |
| *LRIF1* | 0.049 | 0.182 | 2 | hsa-miR-6807-5p | hsa-miR-1234-3p | | | |
| *PCGF3* | 0.047 | 0.178 | 2 | hsa-miR-3180-3p | hsa-miR-3180 | | | |
| *PDE3A* | 0.047 | 0.178 | 2 | hsa-miR-2115-3p | hsa-miR-450a-1-3p | | | |
| *RFC2* | 0.048 | 0.18 | 2 | hsa-miR-6807-5p | hsa-miR-3614-5p | | | |
| *SBF1* | 0.047 | 0.178 | 2 | hsa-miR-3180-3p | hsa-miR-3180 | | | |
| *SUSD1* | 0.049 | 0.182 | 2 | hsa-miR-450a-1-3p | hsa-miR-3614-5p | | | |
| *ZFP69B* | 0.049 | 0.182 | 2 | hsa-miR-6807-5p | hsa-miR-376b-3p | | | |

Analyses were performed using Mienturnet (DB: miRTarBase), and only statistically significant genes ($p < 0.05$) are presented. Gene symbols corresponding to hsa-miR-4516 and hsa-miR-133a-3p, which were upregulated in both the high Hg and GHD groups, are indicated in bold.

**Table S8.** Predicted target genes of differentially expressed miRNAs in the high heavy metal exposure group associated with growth plate regulation, focusing on key pathways of linear growth, including hypothalamic–pituitary–GH/IGF-1 axis signaling, chondrocyte proliferation, hypertrophy, and endochondral ossification.

| **Toxicant group and gene** | **Functional role** | **Endochondral ossification/growth plate (mechanistic link)** | | **Hypothalamic–pituitary–GH/IGF-1 axis (mechanistic link)** |
|---|---|---|---|---|
| **High blood Pb** | | | | |
| ***RUNX3*** | Runt-domain transcription factor; controls cell-cycle and osteo-/chondrogenic differentiation | TGF-β/BMP, NOTCH, WNT/β-catenin, p21/CDKN1A, YAP/TEAD, adherens junctions, thyroid hormone signaling | | None established (indirect endocrine cross-talk via thyroid/retinoid signaling possible) |
| ***MAPK8IP2*** | JNK/p38 scaffold; organizes stress-kinase modules | JNK/p38 MAPK, stress-activated kinase cascades | | None established |
| ***SALL4*** | Zinc-finger transcription factor in embryonic patterning, stemness, limb-bud morphogenesis | WNT/β-catenin, Hedgehog, TGF-β/Smad, PI3K–AKT (via PTEN repression) | | Possible/indirect (GH deficiency reported in a subset of SALL4 disorders; mechanism unclear) |
| ***RXRB*** | Nuclear receptor co-regulator (retinoid/thyroid) | Retinoic acid signaling, thyroid hormone signaling, nuclear receptor/PPAR pathways, PTH axis | | Indirect via thyroid axis; no direct GH signaling |
| ***GNAI2*** | G-protein α-subunit (Giα2); GPCR signal transduction | GPCR–PI3K–AKT, cytoskeletal/adhesion signaling | | *Pituitary mechanism:* Gαi2 mediates SSTR2/5 (Gi/o) inhibition of cAMP–PKA, reducing GH secretion and counteracting GHRH (Gs–cAMP)–driven GH release. |
| ***H2AFX*** | Histone variant; DNA damage signaling/repair | DNA damage response/repair, cell-cycle checkpoints, WNT/β-catenin, NOTCH processing | | None established |

| | | | | |
|---|---|---|---|---|
| ***THSD4 (ADAMTSL6)*** | Extracellular matrix glycoprotein promoting fibrillin-1 microfibrils; modulates TGF-β | TGF-β signaling pathway; ECM-receptor interaction | | None established |
| **High urinary As** | | | | |
| ***ALDH9A1*** | Aldehyde dehydrogenase; aldehyde detoxification; GABA/carnitine metabolism | Mitochondrial metabolism, glycolysis, steroid biosynthesis, p53, IL-17/TNF/NF-κB signaling | | None established |
| ***KPNA6*** | Karyopherin-α6 nuclear import adaptor; shuttles transcription factors to nucleus | Nuclear import, gene expression programs, cell-cycle regulation, developmental signaling | | Putative GH-axis link: facilitates nuclear import of GH-responsive transcription factors (supports GH transcriptional responses) |
| ***NF2 (Merlin)*** | Cytoskeletal–membrane scaffold; tumor suppressor; mechano-transduction | Hippo/YAP, PI3K–AKT, FAK, ERK/MAPK, EGFR | | None established |
| ***PTPN14*** | Non-receptor tyrosine phosphatase; binds YAP; adhesion/lymphangiogenesis | Hippo/YAP, PI3K–AKT/mTOR, TGF-β, NF-κB, cytoskeleton/adhesion | | None established |
| **High urinary Hg** | | | | |
| ***BCL9L*** | Co-activator of β-catenin-TCF transcription; WNT signal amplification | WNT/β-catenin, Hippo cross-talk, TCF-dependent transcription | | None established |
| ***STAT3*** | Cytokine/growth-factor–responsive transcription factor; survival/proliferation/apoptosis | JAK–STAT, gp130/IL-6 family signaling, cytokine signaling, cell-cycle control | | Direct/adjacent: participates in cytokine–JAK–STAT networks intersecting GH signaling (GHR primarily STAT5 but STAT3 modulates GH biology and resistance) |

| | | | | |
|---|---|---|---|---|
| ***FOSL2*** | Transcription factor (Fra-2) forming part of the AP-1 complex; osteo-/chondrogenic transcription | AP-1/TGF-β, osteoclast differentiation, non-canonical WNT, immune regulation | | None established |
| ***MC2R*** | ACTH receptor (GPCR); cortisol synthesis | ACTH–cAMP signaling, adrenal steroid biosynthesis | | Indirect via HPA axis: ACTH–cortisol state modulates GH secretion/action |
| ***ALDH9A1*** | Aldehyde dehydrogenase; aldehyde detoxification; GABA/carnitine metabolism | Mitochondrial metabolism, glycolysis, steroid biosynthesis, p53, IL-17/TNF/NF-κB signaling | | None established |
| ***RFT1*** | ER membrane flippase for lipid-linked oligosaccharides, essential for N-glycosylation | N-glycan biosynthesis; ER protein processing/quality control | | Required for N-glycosylation and trafficking of key surface receptors such as GHR and IGF1R |
| ***TRAF6*** | Adaptor/E3 ubiquitin ligase transmitting innate immune and RANK/RANKL signals to NF-κB/MAPK cascades | TLR/IL-1/TNF signaling; RANK/RANKL (bone remodeling); NF-κB; MAPK (JNK/p38); MyD88 pathways | | None established |
| ***KPNA6*** | Karyopherin-α6 nuclear import adaptor; shuttles transcription factors to nucleus | Nuclear import, gene expression programs, cell-cycle regulation, developmental signaling | | Putative GH-axis link: facilitates nuclear import of GH-responsive transcription factors (supports GH transcriptional responses) |

Relevant pathways were identified using Enrichr (KEGG 2021 Human; Reactome 2024).

**Table S9.** Reported growth-related phenotypes of predicted target genes regulated by differentially expressed miRNAs in the high heavy metal exposure group.

| Gene | Phenotypes associated with growth |
|---|---|
| **Predicted target genes of DEmiRNAs in the high blood Pb group** | |
| ***RUNX3*** | Loss-of-function mutations lead to severe osteopenia, shortened bones, and impaired osteoblast proliferation [49], while gain-of-function possibly inhibited chondrocyte proliferation but accelerated differentiation [50]. *RUNX3* is significantly associated with height in GWAS studies [51]. |
| ***MAPK8IP2*** | 22q13 deletions including *MAPK8IP2* cause short stature and failure to thrive in Phelan-McDermid syndrome [52]. *MAPK8IP2* overexpression can enhance JNK/p38 signaling activation, which may induce premature chondrocyte differentiation and reduced proliferative zones, accelerating hypertrophic differentiation [53]. |
| ***SALL4*** | Heterozygous loss-of-function (LOF) variants underlie Duane-radial-ray / Okihiro syndrome, in which growth impairment is frequently observed [54]. Mouse models with *Sall4* overexpression show mild long bone shortening [55]. Similarly, 20q13.2 duplications involving *SALL4* are associated with skeletal anomalies and growth delay in pediatric cases [56]. *SALL4* is significantly associated with height in GWAS studies [51]. |
| ***RXRB*** | Single-gene knockout (*Rxrb*-/-) mice show mild skeletal phenotype, while compound mutants of multiple RXR subtypes (*Rxrb*-/- plus *Rxrg*+/- or *Rxrg*-/-) demonstrate severe growth defects [57]. Excess *Rxr* activity, including *Rxrb*, accelerates growth plate closure and bone shortening [58]. |
| ***GNAI2*** | Chondrocyte-specific *Gnai2* knockout mice show disrupted growth plate architecture, reduced chondrocyte proliferation, delayed ossification, and shortened long bones [59]. *Gnai2* gain-of function mice model2 exhibit reduced body length, shortened long bones and GH axis dysfunction [60]. GWAS consistently associates *GNAI2* variants with adult height, implicating it in population-level height variation [61]. |
| ***H2AFX*** | *H2afx* loss in mice impairs tissue maintenance and growth under stress, resembling human DNA repair syndromes with short stature [62]. Overexpression of *H2AFX* in humans/mice has not been linked to linear growth. *H2AFX* variants (rs7759, rs7350) show genome-wide associations with height [51]. |
| ***THSD4 (ADAMTSL6)*** | Heterozygous loss-of-function variants in *THSD4* cause marfanoid features [63]. *Thsd4* overexpression in transgenic mice shows excessive fibrillin-1 assembly in cartilage [64] potentially impairing chondrocyte proliferation through TGF-β dysregulation—a hypothesis requiring experimental validation. *THSD4* is significantly associated with height in GWAS studies [51]. |
| **Predicted target genes of DEmiRNAs in the high urine As group** | |
| ***ALDH9A1*** | *Aldh9a1* knockout in combination with *Fanca* causes short stature and developmental defects in mice [35], likely due to aldehyde-induced DNA damage affecting growth plate cells [65]. |

| | |
|---|---|
| ***KPNA6*** | *Kpna6* knockout mice display reduced skeletal growth indicating that loss of *Kpna6* function impairs postnatal bone growth [37]. Given *KPNA6*'s role as an importin-α adaptor for classical nuclear import, disruption of *KPNA6* is mechanistically expected to alter transcriptional programs in growth-plate cells [36]. *KPNA6* is significantly associated with height in GWAS studies [51]. |
| ***NF2*** | *Nf2* deletion in skeletal MSCs impairs Erk1/2 and PI3K/Akt signaling, leading to reduced proliferation, and cranial ossification defects [31]. *NF2* loss disrupts merlin-mediated signaling in growth plate cells, affecting bone growth [66]. *NF2* is significantly associated with height in GWAS studies [67]. |
| ***PTPN14*** | Recessive LOF mutations in *PTPN14* cause congenital lymphedema and short stature [68]. Affected humans and *Ptpn14*-deficient mice both show postnatal growth impairment [69]. Mechanistically, LOF disrupts Hippo pathway signaling and lymphatic function, affecting growth plate activity and tissue homeostasis [30]. *PTPN14* is significantly associated with height in GWAS studies [70]. |
| **Predicted target genes of DEmiRNAs in the high urine Hg group** | |
| ***BCL9L*** | LOF in mice causes shortened long bones, delayed ossification, reduced trabecular bone mass; zebrafish knockdown disrupts craniofacial cartilage [32]. *BCL9L* is significantly associated with height in GWAS studies [51]. |
| ***STAT3*** | LOF in chondrocytes (mouse) causes short stature, growth plate dysfunction, abnormal bone growth [38]; human LOF (autosomal dominant hyper-IgE syndrome) causes skeletal fragility and may reduce growth [71]; GOF in humans causes early-onset short stature and GH resistance [30]; both LOF and GOF can impair endochondral bone growth directly or via inflammation. *STAT3* is significantly associated with height in GWAS studies [51]. |
| ***FOSL2*** | LOF in mice leads to growth retardation, short bones, delayed ossification, reduced matrix, while GOF in mice show increased bone mass and bone formation [34]. *FOSL2* is significantly associated with height in GWAS studies [70]. |
| ***MC2R*** | LOF variants cause familial glucocorticoid deficiency with secondary short stature, hypoglycemia, and immune impairment [73]. *MC2R* is significantly associated with height in GWAS studies [51]. |
| ***ALDH9A1*** | *Aldh9a1* knockout in combination with *Fanca* causes short stature and developmental defects in mice [35], likely due to aldehyde-induced DNA damage affecting growth plate cells [65]. |
| ***RFT1*** | LOF variants cause congenital disorder of glycosylation (RFT1-CDG) with psychomotor delay, sensorineural deficits, seizures, and growth delay [74]; no GOF syndrome reported. *RFT1* is significantly associated with height in GWAS studies [75]. |
| ***TRAF6*** | LOF variants in mice and human show osteopetrosis, shortened long bones, failed tooth eruption, high bone density; mechanism is defective osteoclast-mediated bone remodeling [33,76] |
| ***KPNA6*** | *Kpna6* knockout mice display reduced skeletal growth indicating that loss of *Kpna6* function impairs postnatal bone growth [37]. Given *KPNA6*'s role as an importin-α adaptor for classical nuclear import, disruption of *KPNA6* is mechanistically expected to alter transcriptional programs in growth-plate cells [36]. *KPNA6* is significantly associated with height in GWAS studies [51]. |

## References


1. Benyi, E.; Sävendahl, L. The physiology of childhood growth: Hormonal regulation. *Horm. Res. Paediatr.* **2017**, *88*, 6–14. https://doi.org/10.1159/000471876.
2. Mameli, C.; Guadagni, L.; Orso, M.; Calcaterra, V.; Wasniewska, M.G.; Aversa, T.; Granato, S.; Bruschini, P.; d'Angela, D.; Spandonaro, F.; et al. Epidemiology of growth hormone deficiency in children and adolescents: A systematic review. *Endocrine* **2024**, *85*, 91–98. https://doi.org/10.1007/s12020-024-03778-4.
3. Saltarelli, M.A.; Quarta, A.; Chiarelli, F. Growth plate extracellular matrix defects and short stature in children. *Ann. Pediatr. Endocrinol. Metab.* **2022**, *27*, 247–255. https://doi.org/10.6065/apem.2244120.060.
4. García-Villarino, M.; Signes-Pastor, A.J.; Karagas, M.R.; Riaño-Galán, I.; Rodríguez-Dehli, C.; Grimalt, J.O.; Junqué, E.; Fernández-Somoano, A.; Tardón, A. Exposure to metal mixtures and growth indicators at 4–5 years. A study in the INMA-Asturias cohort. *Environ. Res.* **2022**, *204*, 112375. https://doi.org/10.1016/j.envres.2021.112375.
5. Deierlein, A.L.; Teitelbaum, S.L.; Windham, G.C.; Pinney, S.M.; Galvez, M.P.; Caldwell, K.L.; Jarrett, J.M.; Gajek, R.; Kushi, L.H.; Biro, F.; et al. Lead exposure during childhood and subsequent anthropometry through adolescence in girls. *Environ. Int.* **2019**, *122*, 310–315. https://doi.org/10.1016/j.envint.2018.11.031.
6. La Merrill, M.A.; Vandenberg, L.N.; Smith, M.T.; Goodson, W.; Browne, P.; Patisaul, H.B.; Guyton, K.Z.; Kortenkamp, A.; Cogliano, V.J.; Woodruff, T.J.; et al. Consensus on the key characteristics of endocrine-disrupting chemicals as a basis for hazard identification. *Nat. Rev. Endocrinol.* **2020**, *16*, 45–57. https://doi.org/10.1038/s41574-019-0273-8.
7. Balali-Mood, M.; Naseri, K.; Tahergorabi, Z.; Khazdair, M.R.; Sadeghi, M. Toxic mechanisms of five heavy metals: Mercury, lead, chromium, cadmium, and arsenic. *Front. Pharmacol.* **2021**, *12*, 643972. https://doi.org/10.3389/fphar.2021.643972.
8. Fleisch, A.F.; Burns, J.S.; Williams, P.L.; Lee, M.M.; Sergeyev, O.; Korrick, S.A.; Hauser, R. Blood lead levels and serum insulin-like growth factor 1 concentrations in peripubertal boys. *Environ. Health Perspect.* **2013**, *121*, 854–858. https://doi.org/10.1289/ehp.1206105.
9. Ahmed, S.; Rekha, R.S.; Ahsan, K.B.; Doi, M.; Grandér, M.; Roy, A.K.; Ekström, E.C.; Wagatsuma, Y.; Vahter, M.; Raqib, R. Arsenic exposure affects plasma insulin-like growth factor 1 (IGF-1) in children in rural Bangladesh. *PLoS ONE* **2013**, *8*, e81530. https://doi.org/10.1371/journal.pone.0081530.
10. Chen, A.; Deng, H.; Song, X.; Liu, X.; Chai, L. Effects of separate and combined exposure of cadmium and lead on the endochondral ossification in *Bufo gargarizans*. *Environ. Toxicol. Chem.* **2022**, *41*, 1228–1245. https://doi.org/10.1002/etc.5296.
11. Skalny, A.V.; Aschner, M.; Zhang, F.; Guo, X.; Buha Djordevic, A.; Sotnikova, T.I.; Korobeinikova, T.V.; Domingo, J.L.; Farsky, S.H.P.; Tinkov, A.A. Molecular mechanisms of environmental pollutant-induced cartilage damage: From developmental disorders to osteoarthritis. *Arch. Toxicol.* **2024**, *98*, 2763–2796. https://doi.org/10.1007/s00204-024-03772-9.
12. Gardner, R.M.; Kippler, M.; Tofail, F.; Bottai, M.; Hamadani, J.; Grandér, M.; Nermell, B.; Palm, B.; Rasmussen, K.M.; Vahter, M. Environmental exposure to metals and children's growth to age 5 years: A prospective cohort study. *Am. J. Epidemiol.* **2013**, *177*, 1356–1367. https://doi.org/10.1093/aje/kws437.
13. Malin Igra, A.; Warnqvist, A.; Rahman, S.M.; Ekström, E.C.; Rahman, A.; Vahter, M.; Kippler, M. Environmental metal exposure and growth to 10 years of age in a longitudinal mother–child cohort in rural Bangladesh. *Environ. Int.* **2021**, *156*, 106738. https://doi.org/10.1016/j.envint.2021.106738.
14. Shin, M.W.; Kim, H.B.; Kwon, A.; Park, M.J.; Kim, S.H. Associations between urinary mercury/cadmium concentrations and anthropometric features in Korean children. *Toxics* **2024**, *12*, 175. https://doi.org/10.3390/toxics12030175.
15. Ashley-Martin, J.; Dodds, L.; Arbuckle, T.E.; Lanphear, B.; Muckle, G.; Bouchard, M.F.; Fisher, M.; Asztalos, E.; Foster, W.; Kuhle, S. Blood metal levels and early childhood anthropometric measures in a cohort of Canadian children. *Environ. Res.* **2019**, *179*, 108736. https://doi.org/10.1016/j.envres.2019.108736.
16. Li, M.; Ji, H.; Liu, Y.; Fu, Y.; Lin, W.; Zhu, M.; Xie, D.; Ding, H.; Wang, J. Association between heavy metals exposure and height in Chinese preschoolers. *J. Occup. Environ. Med.* **2023**, *65*, 567–572. https://doi.org/10.1097/JOM.0000000000002834.

17. Cirillo, F.; Catellani, C.; Lazzeroni, P.; Sartori, C.; Street, M.E. The role of microRNAs in influencing body growth and development. *Horm. Res. Paediatr.* **2020**, *93*, 7–15. https://doi.org/10.1159/000504669.
18. Thakore, P.; Delany, A.M. miRNA-based regulation in growth plate cartilage: Mechanisms, targets, and therapeutic potential. *Front. Endocrinol.* **2025**, *16*, 1530374. https://doi.org/10.3389/fendo.2025.1530374.
19. Howe, C.G.; Claus Henn, B.; Farzan, S.F.; Habre, R.; Eckel, S.P.; Grubbs, B.H.; Chavez, T.A.; Faham, D.; Al-Marayati, L.; Lerner, D.; et al. Prenatal metal mixtures and fetal size in mid-pregnancy in the MADRES study. *Environ. Res.* **2021**, *196*, 110388. https://doi.org/10.1016/j.envres.2020.110388.
20. Catellani, C.; Ravegnini, G.; Sartori, C.; Righi, B.; Lazzeroni, P.; Bonvicini, L.; Poluzzi, S.; Cirillo, F.; Predieri, B.; Iughetti, L.; et al. Specific miRNAs change after 3 months of GH treatment and contribute to explain the growth response after 12 months. *Front. Endocrinol.* **2022**, *13*, 896640. https://doi.org/10.3389/fendo.2022.896640.
21. Kim, J.H.; Yun, S.; Hwang, S.S.; Shim, J.O.; Chae, H.W.; Lee, Y.J.; Lee, J.H.; Kim, S.C.; Lim, D.; Yang, S.W.; et al. The 2017 Korean National Growth Charts for children and adolescents: Development, improvement, and prospects. *Korean J. Pediatr.* **2018**, *61*, 135–149. https://doi.org/10.3345/kjp.2018.61.5.135.
22. Afeiche, M.; Peterson, K.E.; Sánchez, B.N.; Schnaas, L.; Cantonwine, D.; Ettinger, A.S.; Solano-González, M.; Hernández-Avila, M.; Hu, H.; Téllez-Rojo, M.M. Windows of lead exposure sensitivity, attained height, and BMI at 48 months. *J. Pediatr.* **2012**, *160*, 1044–1049. https://doi.org/10.1016/j.jpeds.2011.12.022.
23. Ahmadi, S.; Botton, J.; Zoumenou, R.; Ayotte, P.; Fievet, N.; Massougbodji, A.; Alao, M.J.; Cot, M.; Glorennec, P.; Bodeau-Livinec, F. Lead exposure in infancy and subsequent growth in Beninese children. *Toxics* **2022**, *10*, 595. https://doi.org/10.3390/toxics10100595.
24. Burns, J.S.; Williams, P.L.; Lee, M.M.; Revich, B.; Sergeyev, O.; Hauser, R.; Korrick, S.A. Peripubertal blood lead levels and growth among Russian boys. *Environ. Int.* **2017**, *106*, 53–59. https://doi.org/10.1016/j.envint.2017.05.023.
25. Saha, K.K.; Engström, A.; Hamadani, J.D.; Tofail, F.; Rasmussen, K.M.; Vahter, M. Pre- and postnatal arsenic exposure and body size to 2 years of age: A cohort study in rural Bangladesh. *Environ. Health Perspect.* **2012**, *120*, 1208–1214. https://doi.org/10.1289/ehp.1003378.
26. Ma, J.; Geng, S.; Sun, Q.; Zhang, X.; Han, L.; Yao, X.; Zhang, B.; Zhu, L.; Wen, J. Exposure to metal mixtures and young children's growth and development: A biomonitoring-based study in Eastern China. *Ecotoxicol. Environ. Saf.* **2023**, *268*, 115726. https://doi.org/10.1016/j.ecoenv.2023.115726.
27. EFSA Panel on Contaminants in the Food Chain (CONTAM); Schrenk, D.; Bignami, M.; Bodin, L.; Chipman, J.K.; del Mazo, J.K.; Grasl-Kraupp, B.; Hogstrand, C.; Hoogenboom, L.; Hoogenboom, L.R.; et al. Update of the risk assessment of inorganic arsenic in food. *EFSA J.* **2024**, *22*, e8488. https://doi.org/10.2903/j.efsa.2024.8488.
28. Jedrychowski, W.A.; Perera, F.P.; Majewska, R.; Mrozek-Budzyn, D.; Mroz, E.; Roen, E.L.; Sowa, A.; Jacek, R. Depressed height gain of children associated with intrauterine exposure to polycyclic aromatic hydrocarbons (PAH) and heavy metals: The cohort prospective study. *Environ. Res.* **2015**, *136*, 141–147. https://doi.org/10.1016/j.envres.2014.08.047.
29. Ben-Shlomo, A.; Melmed, S. Pituitary somatostatin receptor signaling. *Trends Endocrinol. Metab.* **2010**, *21*, 123–133. https://doi.org/10.1016/j.tem.2009.12.003.
30. Wang, W.; Huang, J.; Wang, X.; Yuan, J.; Li, X.; Feng, L.; Park, J.I.; Chen, J. PTPN14 is required for the density-dependent control of YAP1. *Genes Dev.* **2012**, *26*, 1959–1971. https://doi.org/10.1101/gad.192955.112.
31. Liao, J.; Huang, Y.; Sun, F.; Zheng, C.; Yao, Y.; Zhang, C.; Zhou, C.; Zhang, X.; Wu, M.; Chen, G. Nf2–FAK signaling axis is critical for cranial bone ossification and regeneration. *Nat. Commun.* **2025**, *16*, 2478. https://doi.org/10.1038/s41467-025-57808-4.
32. Cantù, C.; Felker, A.; Zimmerli, D.; Prummel, K.D.; Cabello, E.M.; Chiavacci, E.; Méndez-Acevedo, K.M.; Kirchgeorg, L.; Burger, S.; Ripoll, J.; et al. Mutations in *Bcl9* and *Pygo* genes cause congenital heart defects by tissue-specific perturbation of Wnt/β-catenin signaling. *Genes Dev.* **2018**, *32*, 1443–1458. https://doi.org/10.1101/gad.315531.118.
33. Lomaga, M.A.; Yeh, W.C.; Sarosi, I.; Duncan, G.S.; Furlonger, C.; Ho, A.; Morony, S.; Capparelli, C.; Van, G.; Kaufman, S.; et al. TRAF6 deficiency results in osteopetrosis and defective interleukin-1, CD40, and LPS signaling. *Genes Dev.* **1999**, *13*, 1015–1024. https://doi.org/10.1101/gad.13.8.1015.

34. Bozec, A.; Bakiri, L.; Jimenez, M.; Schinke, T.; Amling, M.; Wagner, E.F. Fra-2/AP-1 controls bone formation by regulating osteoblast differentiation and collagen production. *J. Cell Biol.* **2010**, *190*, 1093–1106. https://doi.org/10.1083/jcb.201002111.
35. Jung, M.; Kim, J.; Park, Y.; Ilyashov, I.; Yang, F.; Choijilsuren, H.B.; Keahi, D.; Durmaz, J.A.; Bea, H.; Goldfarb, A.M.; et al. ALDH9A1 deficiency as a source of endogenous DNA damage that requires repair by the Fanconi anemia pathway. *J. Cell Biol.* **2025**, *224*, e202407141. https://doi.org/10.1083/jcb.202407141.
36. Diaz, C.; Thankam, F.G.; Agrawal, D.K. Karyopherins in the remodeling of extracellular matrix: Implications in tendon injury. *J. Orthop. Sports Med.* **2023**, *5*, 357–374. https://doi.org/10.26502/josm.511500122.
37. Liu, N.; Qadri, F.; Busch, H.; Huegel, S.; Sihn, G.; Chuykin, I.; Hartmann, E.; Bader, M.; Rother, F. *Kpna6* deficiency causes infertility in male mice by disrupting spermatogenesis. *Development* **2021**, *148*, dev198374. https://doi.org/10.1242/dev.198374.
38. Liu, N.Q.; Lin, Y.; Li, L.; Lu, J.; Geng, D.; Zhang, J.; Jashashvili, T.; Buser, Z.; Magallanes, J.; Tassey, J.; et al. Gp130/STAT3 signaling is required for homeostatic proliferation and anabolism in postnatal growth plate and articular chondrocytes. *Commun. Biol.* **2022**, *5*, 64. https://doi.org/10.1038/s42003-021-02944-y.
39. Mazziotti, G.; Giustina, A. Glucocorticoids and the regulation of growth hormone secretion. *Nat. Rev. Endocrinol.* **2013**, *9*, 265–276. https://doi.org/10.1038/nrendo.2013.5.
40. Di Patria, L.; Annibalini, G.; Morrone, A.; Ferri, L.; Saltarelli, R.; Galluzzi, L.; Diotallevi, A.; Bocconcelli, M.; Donati, M.A.; Barone, R.; et al. Defective IGF-1 . signaling activation in congenital disorders of glycosylation. *Cell. Mol. Life Sci.* **2022**, *79*, 150. https://doi.org/10.1007/s00018-022-04180-x.
41. Zhang, Y.; Xie, R.-L.; Croce, C.M.; Stein, J.L.; Lian, J.B.; van Wijnen, A.J.; Stein, G.S. A program of microRNAs controls osteogenic lineage progression by targeting transcription factor Runx2. *Proc. Natl. Acad. Sci.* **2011**, 108, 9863–9868. https://doi.org/10.1073/pnas.1018493108.
42. Zhang, P.; Chen, F.-Z.; Jia, Q.-B.; Hu, D.-F. Upregulation of microRNA-133a and downregulation of connective tissue growth factor suppress cell proliferation, migration, and invasion in human glioma through the JAK/STAT signaling pathway. *IUBMB Life* **2019**, 71, 1857–1875. https://doi.org/10.1002/iub.2126.
43. Chen, G.; Wang, L.; Liu, X.; Su, H.; Wang, P.; Li, J.; Yu, S.; Chen, Y. MiR-133a inhibits cell proliferation and invasion by targeting IGF-1R in osteosarcoma. *Cell Physiol. Biochem.* **2016**, 38, 598–608. https://doi.org/10.1159/000438653.
44. Chowdhari, S.; Saini, N. hsa-miR-4516 Mediated downregulation of STAT3/CDK6/UBE2N plays a role in PUVA induced apoptosis in keratinocytes. *J. Cell. Physiol.* **2014**, 229, 1630–1638. https://doi.org/10.1002/jcp.24608.
45. Chowdhari, S.; Sardana, K.; Saini, N. MiR-4516, a microRNA downregulated in psoriasis, inhibits keratinocyte motility by targeting fibronectin/integrin α9 signaling. *Biochim. Biophys. Acta Mol. Basis Dis.* **2017**, 1863, 3142–3152. https://doi.org/10.1016/j.bbadis.2017.08.014.
46. Mandourah, A.Y.; Ranganath, L.R.; Barraclough, R.; Vinjamuri, S.; Van't Hof, R.; Hamill, S.; Czanner, G.; Dera, A.A.; Wang, D.; Barraclough, D.L. Circulating microRNAs as potential diagnostic biomarkers for osteoporosis. *Sci. Rep.* **2018**, 8, 8421. https://doi.org/10.1038/s41598-018-26525-y.
47. Shen, N.; Tang, L.; Qian, Y.; Pan, J.; Pan, J.; Miao, H.; Zhang, H.; Fang, H.; Yu, X.; Xing, L. Serum miR-4488 as a potential biomarker of lean nonalcoholic fatty liver disease. *Ann. Transl. Med.* **2023**, 11, 173. https://doi.org/10.21037/atm-22-6620.
48. Conesa, A.; Madrigal, P.; Tarazona, S.; Gomez-Cabrero, D.; Cervera, A.; McPherson, A.; Szcześniak, M.W.; Gaffney, D.J.; Elo, L.L.; Zhang, X.; Mortazavi, A. A survey of best practices for RNA-seq data analysis. *Genome Biol.* **2016**, 17, 13. https://doi.org/10.1186/s13059-016-0881-8.
49. Bauer, O.; Sharir, A.; Kimura, A.; Hantisteanu, S.; Takeda, S.; Groner, Y. Loss of osteoblast Runx3 produces severe congenital osteopenia. *Mol. Cell. Biol.* **2015**, *35*, 1097–1109. https://doi.org/10.1128/MCB.01106-14.
50. Wang, Y.; Feng, Q.; Ji, C.; Liu, X.; Li, L.; Luo, J. RUNX3 plays an important role in mediating the BMP9-induced osteogenic differentiation of mesenchymal stem cells. *Int. J. Mol. Med.* **2017**, *40*, 1991–1999. https://doi.org/10.3892/ijmm.2017.3155.
51. Yengo, L.; Vedantam, S.; Marouli, E.; Sidorenko, J.; Bartell, E.; Sakaue, S.; Graff, M.; Eliasen, A.U.; Jiang, Y.; Raghavan, S.; et al. A saturated map of common genetic variants associated with human height. *Nature* **2022**, *610*, 704–712. https://doi.org/10.1038/s41586-022-05275-y.

52. Frye, R.E.; Cox, D.; Slattery, J.; Tippett, M.; Kahler, S.; Granpeesheh, D.; Damle, S.; Legido, A.; Goldenthal, M.J. Mitochondrial dysfunction may explain symptom variation in Phelan–McDermid syndrome. *Sci. Rep.* **2016**, *6*, 19544. https://doi.org/10.1038/srep19544.
53. Stanton, L.-A.; Sabari, S.; Sampaio, A.V.; Underhill, T.M.; Beier, F. p38 MAP kinase signalling is required for hypertrophic chondrocyte differentiation. *Biochem. J.* **2004**, *378*, 53–62. https://doi.org/10.1042/BJ20030874.
54. Ma, X.; Huang, R.; Li, G.; Zhang, T.; Ma, J. A de novo mutation of *SALL4* in a Chinese family with Okihiro syndrome. *Mol. Med. Rep.* **2022**, *25*, 131. https://doi.org/10.3892/mmr.2022.12647.
55. Chen, K.Q.; Anderson, A.; Kawakami, H.; Kim, J.; Barrett, J.; Kawakami, Y. Normal embryonic development and neonatal digit regeneration in mice overexpressing a stem cell factor, Sall4. *PLoS ONE* **2022**, *17*, e0267273. https://doi.org/10.1371/journal.pone.0267273.
56. Briand-Suleau, A.; Martinovic, J.; Tosca, L.; Tou, B.; Brisset, S.; Bouligand, J.; Delattre, V.; Giurgea, I.; Bachir, J.; Folliot, P.; et al. SALL4 and NFATC2: Two major actors of interstitial 20q13.2 duplication. *Eur. J. Med. Genet.* **2014**, *57*, 174–180. https://doi.org/10.1016/j.ejmg.2013.12.013.
57. Krezel, W.; Dupé, V.; Mark, M.; Dierich, A.; Kastner, P.; Chambon, P. RXR gamma null mice are apparently normal and compound RXR alpha +/−/RXR beta −/−/RXR gamma −/− mutant mice are viable. *Proc. Natl. Acad. Sci. USA* **1996**, *93*, 9010–9014. https://doi.org/10.1073/pnas.93.17.9010.
58. Dupuis, H.; Pest, M.A.; Hadzic, E.; Vo, T.X.; Hardy, D.B.; Beier, F. Exposure to the RXR agonist SR11237 in early life causes disturbed skeletal morphogenesis in a rat model. *Int. J. Mol. Sci.* **2019**, *20*, 5198. https://doi.org/10.3390/ijms20205198.
59. Plummer, N.W.; Spicher, K.; Malphurs, J.; Akiyama, H.; Abramowitz, J.; Nürnberg, B.; Birnbaumer, L. Development of the mammalian axial skeleton requires signaling through the G$\alpha$i subfamily of heterotrimeric G proteins. *Proc. Natl. Acad. Sci. USA* **2012**, *109*, 21366–21371. https://doi.org/10.1073/pnas.1219810110.
60. Omouessi, S.T.; Leipprandt, J.R.; Akoume, M.-Y.; Charbeneau, R.; Wade, S.; Neubig, R.R. Mice with an RGS-insensitive G$\alpha$i2 protein show growth hormone axis dysfunction. *Mol. Cell. Endocrinol.* **2021**, *521*, 111098. https://doi.org/10.1016/j.mce.2020.111098.
61. Yengo, L.; Sidorenko, J.; Kemper, K.E.; Zheng, Z.; Wood, A.R.; Weedon, M.N.; Frayling, T.M.; Hirschhorn, J.; Yang, J.; Visscher, P.M. Meta-analysis of genome-wide association studies for height and body mass index in ~700,000 individuals of European ancestry. *Hum. Mol. Genet.* **2018**, *27*, 3641–3649. https://doi.org/10.1093/hmg/ddy271.
62. Celeste, A.; Petersen, S.; Romanienko, P.J.; Fernandez-Capetillo, O.; Chen, H.T.; Sedelnikova, O.A.; Reina-San-Martin, B.; Coppola, V.; Meffre, E.; Difilippantonio, M.J.; et al. Genomic instability in mice lacking histone H2AX. *Science* **2002**, *296*, 922–927. https://doi.org/10.1126/science.1069398.
63. Arnaud, P.; Mougin, Z.; Boileau, C.; Le Goff, C. Cooperative mechanism of ADAMTS/ADAMTSL and fibrillin-1 in Marfan syndrome and acromelic dysplasias. *Front. Genet.* **2021**, *12*, 734718. https://doi.org/10.3389/fgene.2021.734718.
64. Tsutsui, K.; Manabe, R.; Yamada, T.; Nakano, I.; Oguri, Y.; Keene, D.R.; Sengle, G.; Sakai, L.Y.; Sekiguchi, K. ADAMTSL-6 is a novel extracellular matrix protein that binds to fibrillin-1 and promotes fibrillin-1 fibril formation. *J. Biol. Chem.* **2010**, *285*, 4870–4882. https://doi.org/10.1074/jbc.M109.076919.
65. Blouin, T.; Saini, N. Aldehyde-induced DNA–protein crosslinks: DNA damage, repair and mutagenesis. *Front. Oncol.* **2024**, *14*, 1478373. https://doi.org/10.3389/fonc.2024.1478373.
66. Wu, S.; Bian, Y.; Zhang, C.; Liu, K.; Sun, F.; Chen, E.; Zhou, C.; Yu, L.; Chen, G.; Wu, M. Merlin controls limb development and thumb formation by regulating primary cilium–Hedgehog signaling. *Cell Rep.* **2025**, *44*, 115849. https://doi.org/10.1016/j.celrep.2025.115849.
67. Ramírez-Luzuriaga, M.J.; Kobes, S.; Hsueh, W.-C.; Baier, L.J.; Hanson, R.L. Novel signals and polygenic score for height are associated with pubertal growth traits in Southwestern American Indians. *Hum. Mol. Genet.* **2024**, *33*, 981–990. https://doi.org/10.1093/hmg/ddae030.
68. Bordbar, A.; Maroofian, R.; Ostergaard, P.; Kashaki, M.; Nikpour, S.; Gordon, K.; Crosby, A.; Khosravi, P.; Shojaei, A. A homozygous loss-of-function mutation in *PTPN14* causes a syndrome of bilateral choanal atresia and early infantile-onset lymphedema. *Meta Gene* **2017**, *14*, 53–58. https://doi.org/10.1016/j.mgene.2017.07.006.

69. Au, A.C.; Hernandez, P.A.; Lieber, E.; Nadroo, A.M.; Shen, Y.-M.; Kelley, K.A.; Gelb, B.D.; Diaz, G.A. Protein tyrosine phosphatase PTPN14 is a regulator of lymphatic function and choanal development in humans. *Am. J. Hum. Genet.* **2010**, *87*, 436–444. https://doi.org/10.1016/j.ajhg.2010.08.008.
70. Kichaev, G.; Bhatia, G.; Loh, P.-R.; Gazal, S.; Burch, K.; Freund, M.K.; Schoech, A.; Pasaniuc, B.; Price, A.L. Leveraging polygenic functional enrichment to improve GWAS power. *Am. J. Hum. Genet.* **2019**, *104*, 65–75. https://doi.org/10.1016/j.ajhg.2018.11.008.
71. Sowerwine, K.J.; Shaw, P.A.; Gu, W.; Ling, J.C.; Collins, M.T.; Darnell, D.N.; Anderson, V.L.; Davis, J.; Hsu, A.; Welch, P.; et al. Bone density and fractures in autosomal dominant hyper IgE syndrome. *J. Clin. Immunol.* **2014**, *34*, 260–264. https://doi.org/10.1007/s10875-013-9982-2.
72. Vogel, T.P.; Leiding, J.W.; Cooper, M.A.; Forbes Satter, L.R. STAT3 gain-of-function syndrome. *Front. Pediatr.* **2023**, *10*, 770077. https://doi.org/10.3389/fped.2022.770077.
73. Lin, L.; Hindmarsh, P.C.; Metherell, L.A.; Alzyoud, M.; Al-Ali, M.; Brain, C.E.; Clark, A.J.L.; Dattani, M.T.; Achermann, J.C. Severe loss-of-function mutations in the adrenocorticotropin receptor (ACTHR, MC2R) can be found in patients diagnosed with salt-losing adrenal hypoplasia. *Clin. Endocrinol.* **2007**, *66*, 205–210. https://doi.org/10.1111/j.1365-2265.2006.02709.x.
74. Vleugels, W.; Haeuptle, M.A.; Ng, B.G.; Michalski, J.-C.; Battini, R.; Dionisi-Vici, C.; Ludman, M.D.; Jaeken, J.; Foulquier, F.; Freeze, H.H.; et al. RFT1 deficiency in three novel CDG patients. *Hum. Mutat.* **2009**, *30*, 10.1002/humu.21085. https://doi.org/10.1002/humu.21085.
75. Berndt, S.I.; Gustafsson, S.; Mägi, R.; Ganna, A.; Wheeler, E.; Feitosa, M.F.; Justice, A.E.; Monda, K.L.; Croteau-Chonka, D.C.; Day, F.R.; et al. Genome-wide meta-analysis identifies 11 new loci for anthropometric traits and provides insights into genetic architecture. *Nat. Genet.* **2013**, *45*, 501–512. https://doi.org/10.1038/ng.2606.
76. Weisz Hubshman, M.; Basel-Vanagaite, L.; Krauss, A.; Konen, O.; Levy, Y.; Garty, B.Z.; Smirin-Yosef, P.; Maya, I.; Lagovsky, I.; Taub, E.; et al. Homozygous deletion of *RAG1*, *RAG2* and the 5′ region of *TRAF6* causes severe immune suppression and atypical osteopetrosis. *Clin. Genet.* **2017**, *91*, 902–907. https://doi.org/10.1111/cge.12916
